# The structural basis of the pH-homeostasis mediated by the Cl^−^/HCO_3_^−^ exchanger, AE2

**DOI:** 10.1038/s41467-023-37557-y

**Published:** 2023-03-31

**Authors:** Qing Zhang, Liyan Jian, Deqiang Yao, Bing Rao, Ying Xia, Kexin Hu, Shaobai Li, Yafeng Shen, Mi Cao, An Qin, Jie Zhao, Yu Cao

**Affiliations:** 1 Department of Orthopaedics, Shanghai Key Laboratory of Orthopaedic Implant, Shanghai Ninth People’s Hospital, Shanghai Jiao Tong University School of Medicine, 200011 Shanghai, China; 2 Institute of Precision Medicine, the Ninth People’s Hospital, Shanghai Jiao Tong University School of Medicine, 115 Jinzun Road, 200125 Shanghai, China; 3 Department of Orthopaedics, Shanghai Frontiers Science Center of Degeneration and Regeneration in Skeletal System, Shanghai Ninth People’s Hospital, Shanghai Jiao Tong University School of Medicine, 200011 Shanghai, China; 4 State Key Laboratory of Oncogenes and Related Genes, Ren Ji Hospital, Shanghai Jiao Tong University School of Medicine, 200127 Shanghai, China

**Keywords:** Cryoelectron microscopy, Membrane proteins, Permeation and transport

## Abstract

The cell maintains its intracellular pH in a narrow physiological range and disrupting the pH-homeostasis could cause dysfunctional metabolic states. Anion exchanger 2 (AE2) works at high cellular pH to catalyze the exchange between the intracellular HCO_3_^−^ and extracellular Cl^−^, thereby maintaining the pH-homeostasis. Here, we determine the cryo-EM structures of human AE2 in five major operating states and one transitional hybrid state. Among those states, the AE2 shows the inward-facing, outward-facing, and intermediate conformations, as well as the substrate-binding pockets at two sides of the cell membrane. Furthermore, critical structural features were identified showing an interlock mechanism for interactions among the cytoplasmic N-terminal domain and the transmembrane domain and the self-inhibitory effect of the C-terminal loop. The structural and cell-based functional assay collectively demonstrate the dynamic process of the anion exchange across membranes and provide the structural basis for the pH-sensitive pH-rebalancing activity of AE2.

## Abstract (web summary)

Cells maintain a narrow physiological pH by exchanging intracellular bicarbonate for extracellular chloride. Here, authors determine the cryo-EM structures of human anion exchanger 2 (AE2) in five major operating states and one transitional state, to collectively demonstrate the process of pH-balancing.

## Introduction

The cell strictly controls the intracellular pH (pHi) to maintain the functions of macromolecules and organelles. The intracellular pH could shift from its normal value upon the energy metabolism, as well as some cellular activities such as acid secretion, and thus the recovery of physiological pH is essential for cell functions. In addition to the direct control of the H^+^ level by the proton pumps, two of the most abundant anions in the human body, chloride and bicarbonate (HCO_3_^−^), are mainly used to adjust the acid-base homeostasis. For example, the bicarbonate efflux/chloride influx could decrease the pH_i_ to protect the acid-secreting cells from intracellular alkalinization^1–3^. Both Cl^−^ and HCO_3_^−^ ions are membrane impermeable, and two solute carrier families, SLC4A and SLC26A, were reported responsible for HCO_3_^−^ transmembrane transportation^4–6^. There are ten SLC4A members (SLC4A1-5 and 7–11) in the human genome, and nine of them were reported to mediate HCO_3_^−^ transport, with SLC4A11 reported as a Na^+^-coupled B(OH)_4_^−^ cotransporter^7,8^. The SLC4A could be classified into two functional subfamilies: sodium-coupled HCO_3_^−^ transporters (NCBTs) and sodium-independent HCO_3_^−^/Cl^−^ transporters, the latter including three anion exchangers, AE1-3, encoded by SLC4A1-3, respectively^6,9^. AEs catalyze the anion exchange between HCO_3_^−^ and Cl^−^ across the plasma membrane, thereby regulating the intracellular pH homeostasis, Cl^−^ concentration, and cell volume^7,10^. While the AE1 and AE3 function in the restricted tissues with expression, AE2 is widely distributed in basolateral membranes of most epithelial cells and thus considered a housekeeping HCO_3_^−^ transporter^7,11^. Highly expressed in gastric parietal cells, intestinal enterocytes, respiratory epithelium, and biliary ductular epithelium^12,13^, AE2 mediates HCO_3_^−^ efflux in exchange for extracellular Cl^−^ to avoid intracellular alkalization, which is essential to sustain the fundamental function of acid-secreting cells^3,14,15^. AE2 was first cloned in 1986 as a non-erythroid band 3-like protein^16^. It was spliced into three major AE2 isoforms where AE2a is extensively expressed, and AE2b is highly expressed in the lung, liver, kidney, and gastrointestinal tract, with AE2c exclusively found in gastric cells^17,18^. AE2 is up-regulation during osteoclast differentiation, spermatogenesis, and enamel maturation^3,19,20^, and the dysfunction of AE2 is associated with achlorhydria, osteopetrosis, male infertile, and primary biliary cirrhosis^3,6,19,21–23^, showing the importance of AE2 in cellular pH homeostasis.

AE2 catalyzes Cl^−^/HCO_3_^−^ exchange in response to elevated pH_i_ and hypertonicity^24,25^, which could be inhibited by the canonical anion transporter inhibitors DIDS and H2DIDS^6^. In acid-secreting cells such as gastric cells and osteoclasts, AE2 was localized in the basolateral membrane where it imports Cl^−^ to compensate for the Cl^−^ loss during the acid secretion at the apical side and export HCO_3_^−^ to lower the elevated pH upon the HCl secretion, thereby protecting cells from cytosolic alkalosis^3,26^. Although the role of AE2 in Cl^−^/HCO_3_^−^ exchange and cellular pH homeostasis has been established, the pH-sensing and the pH-dependent anion transportation mechanisms are yet to be elucidated. Previous structural studies on human AE1, an anion exchanger with low pH dependence, revealed a dimeric architecture for AEs, and all the structures were solved in an outward-facing conformation^27–29^, except a recent report on the inward-facing conformation of bovine AE1 at a limited resolution ranging from 4.2 to 6.9 Å^30^, leaving the inward-facing and intermediate conformations yet to be precisely defined. Therefore, the dynamic process during anions transportation and the pH regulation mechanism of Cl^−^/HCO_3_^−^ exchangers remain unclear. Here, we determine the cryo-EM structures of human AE2 as full-length protein and membrane-bound exchanger domain in its inward-facing, outward-facing, and intermediate conformations. The structures and the functional assays on the pH-adjustment activities of AE2 suggest a pH-sensor role of the NTD of AE2, and the pH shift-induced conformational change triggers the dislocations of the transmembrane helices to exchange the anions across the membrane.

## Results

### The cryo-electron microscopy analysis of human AE2

**Fig. 1:**
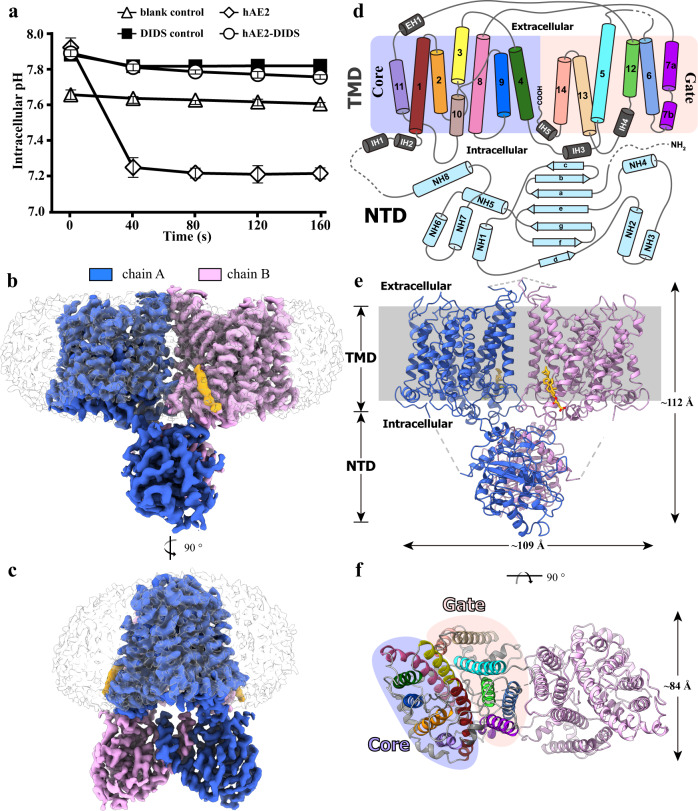
The cryo-EM structure of human AE2 in an inward-facing conformation. **a** The cell-based function assay on hAE2. The intracellular pH changes upon the recovery of extracellular Cl^−^ were recorded for the Expi293 cells overexpressing the human SLC4A2 gene in the presence or absence of DIDS inhibitor, with the cells without overexpression as the blank control. The results were shown as mean ± s. d. of experiments in triplicate. **b**, **c** hAE2^acidic-KNO3^ cryo-EM map as viewed parallel to the cell membrane. The electron density corresponding to two protomers in dimeric hAE2 was shown in blue (chain A) and pink (chain B), respectively. The non-protein electron density surrounding the transmembrane regions (TM) was shown in gray, except for the electron density of CHS, colored in orange. **d** The cartoon representation of hAE2 monomer topology. **e** The dimeric structure of the hAE2. The hAE2 structure was shown as cartoon model and viewed at the same angle as (**b**). The membrane region was shown as gray background. **f** The cartoon model of the hAE2 dimer as viewed from the extracellular side of the cell membrane. Protomer B was colored in pink, and protomer A was colored to the same scheme as shown in (**d**).

The three isoforms of human AE2 (hAE2) were over-expressed in Expi293 cells with comparable yields (Supplementary Fig. 1a, b). When the cells pre-equilibrated in a low [Cl^−^] buffer were replenished with physiological [Cl^−^], the hAE2-overexpressing cells showed a pH decrease resulting from the exchange of extracellular Cl^−^ and intracellular HCO_3_^−^, a process abolished upon the treatment with DIDS (4,4′-Diisothiocyanatostilbene-2,2′-disulfonate), a broad-spectrum anion transporter inhibitor (Fig. 1a, Supplementary Fig. 1c, d). In addition to the pH decrease effects, higher resting intracellular pHs (pHs at time 0 of the assay) were also noticed in the cell overexpressing functional hAE2 compared with those without hAE2 overexpression or treated with DIDS (Fig. 1a and Supplementary Fig. 1d). This higher cellular pHs upon the low Cl^−^-pretreatment suggest a bi-directional transportation by AE2: while mediating a Cl^−^ influx/HCO_3_^−^ efflux at high extracellular Cl^−^ conditions (e.g., in stomach or bone resorption lacuna), AE2 can also conduct a reverse anion exchange at low extracellular Cl^−^ conditions, i.e., Cl^−^ efflux/HCO_3_^−^ influx, thereby elevating the cellular pHs (Supplementary Fig. 1c). Among various hAE2 constructs tested for overexpression, the AE2^177–1241^ (named hAE2 hereafter) showed the best stability and homogeneity as evaluated by size-exclusion chromatography (SEC) and EM analysis (Supplementary Fig. 1e). The hAE2 protein was solubilized from the cell membrane using lauryl maltose neopentyl glycol (LMNG) as detergent and further purified with SEC using glyco-diosgenin (GDN) as the detergent in the mobile phase. Cryo-EM images of hAE2 were collected using a Titan Krios transmission electron microscope (FEI) operated at 300 kV, and data processing was performed using RELION-3 and cryoSPARC^31,32^. Human AE2 showed a good performance in SEC in the presence of NO3^−^ or Cl^−^ at a near-physiological pH (7.25) and forms a stable homodimer in purification and EM analysis (Supplementary Figs. 1f, 2, 3), consistent with the structural studies on AE1. Both 2D- and 3D classification on the particles of hAE2 purified in solutions supplemented with NaHCO_3_ showed strong signals corresponding to the membrane-bound domains with weak and discontinuous electron density for the cytoplasmic NTD (Supplementary Fig. 4). Similar results were observed when hAE2 was purified in a NaHCO_3_-free solution of pH 8.32, and AE2 deprived of chloride/bicarbonate at basic pHs in two conformations (hAE2_inter_^basic-KNO3^, hAE2_out_^basic-KNO3^), with resolutions of 3.25 Å, 3.09 Å, respectively (Supplementary Fig. 5). To stabilize hAE2, we further purified it in the KNO_3_ solutions free of chloride/bicarbonate at lower pHs (hereinafter hAE2^acidic-KNO3^), and the EM analysis showed a complete electron map containing both membrane-bound and cytoplasmic domain at pHs ranging from 7.25 to 6.55 (Supplementary Figs. 2, 6). Further refinement and polishing generated final EM maps with an overall resolution of 3.32 Å for hAE2^acidic-KNO3^ in full-length and 2.89 Å for hAE2^NaHCO3^ as a membrane-bound domain only (Supplementary Figs. 2, 4). The molecular models were thus built in the corresponding EM maps (Fig. 1b–d, and Supplementary Fig. 8a–d), respectively, using the crystal structure of human AE1 as the starting model. Using similar methods, we further determined EM structures for hAE2 supplemented with inhibitor DIDS (hAE2^DIDS^) in the presence of HCO_3_^−^, and two maps in different conformations were reconstructed, one is named hAE2^DIDS^ with C2 symmetry, the other is hAE2^asymmetry^ with C1 symmetry, at resolutions of 3.08 Å and 3.17 Å respectively (Supplementary Fig. 7 and Supplementary Table 1).

### The overall structure of human AE2 in full length

**Fig. 2:**
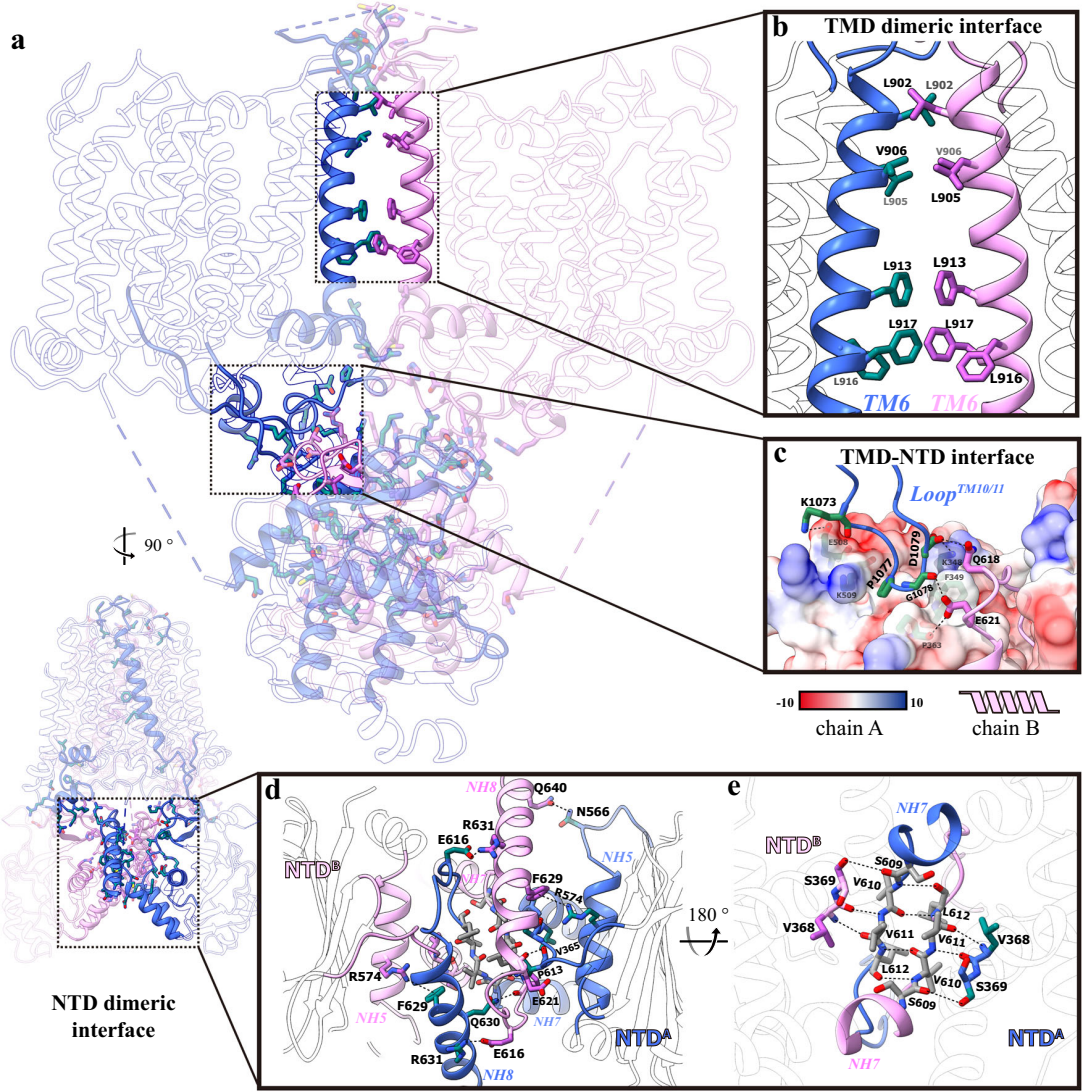
The dimerization of hAE2. **a** The secondary structures of hAE2 involved with dimerization. The cartoon model of hAE2^acidic-KNO3^ was viewed at two angles, and the secondary structures at the dimeric interface were highlighted in blue and pink for protomers A and B, respectively. **b** The dimeric interactions in TMD. The TMD dimeric interface was formed by the TM6 helices from protomer A (blue) and B (pink). The residues mediating the dimerization were shown as stick model and colored by elements**. c** The NTD-TMD interactions. The NTD of protomer A was shown as calculated solvent-accessible electrostatic surface–potential maps. The long loop between TM10 and 11 (loop^TM10/11^) of protomer A and the NTD of protomer B were shown as cartoon model and colored in blue and pink, respectively. The interacting residues were shown as stick model and colored by elements. **d**, **e** The NTD dimeric interface viewed at two angles. The NTDs of protomer A and B were shown as cartoon models and colored in blue and pink, respectively. The interacting residues were shown as stick model and colored by elements.

Overall, hAE2^acidic-KNO3^ folds into a twisted homodimer with the dimeric interface of the transmembrane domain (TMD) perpendicular to that of the N-terminal domain (NTD) (Fig. 1e). The electron density was fragmentary for amino acid 1–319, 433–498, 643–672 and 856–893 and thus remains unmodeled in our structures, which represent the disordered N-terminal, the hinge domain between NTD and TMD, and disorder extracellular loop at the dimeric interface, respectively (Fig. 1e, f and Supplementary Fig. 9). Similar to other members in the SLC4A family, the hAE2 TMD is comprised of 14 transmembrane helices (TM1-14) and adapts to a reported UraA fold^33^, where TM1-4, 8–11 form the “Core” domain and TM 5–7, 12–14 form the “Gate” domain (Fig. 1d). The NTD of hAE2 is located in the cytoplasm and comprised with seven β-strands (a-g) sandwiched by eight α-helices (NH1-8) (Fig. 1d, e). Both the TMD and NTD involved in dimerization of hAE2 (Fig. 2a). The dimer was majorly maintained by the TMD dimeric interface (Fig. 2b) and the NTD dimeric interface (Fig. 2d, e), where the NH5 and NH7-loop-NH8 form a tightly interacting network with a series of salt bridges and hydrogen bonds. Furthermore, an intramolecular interaction was observed at the interface between TMD and NTD. The long, flexible loop between TM10 and 11 (loop^TM10/11^) extends from the TMD and reaches the surface of the NTD of the same protomer (Fig. 2a, c) where it meets helix NH4 and strands b/c, as well as the loop between NH7 and 8 of the neighboring protomer, suggesting a role of loop^TM10/11^ in converting the conformation changes in NTD to the transmembrane helices. The TMD-NTD contact is stabilized by the intramolecular electrostatic interactions among K1073-E508 and D1079-K348, as well as the intermolecular hydrogen bonds among G1078-E621 and D1079-Q618. One long blob of non-proteinous electron density was identified on the surface of TMD near helices TM13 and IH4 and fit well with the cholesteryl hemisuccinate (CHS) supplemented during purification (Supplementary Fig. 10a). The CHS molecule binds with the TMD with the hydrophobic cholesterol moiety deeply embedded in the membrane region, and the carboxyl group of the hemisuccinate stabilized with its interaction with R1162 (Supplementary Fig. 10b).

### The inward-facing anion-binding pocket and the gating C-terminal domain

**Fig. 3:**
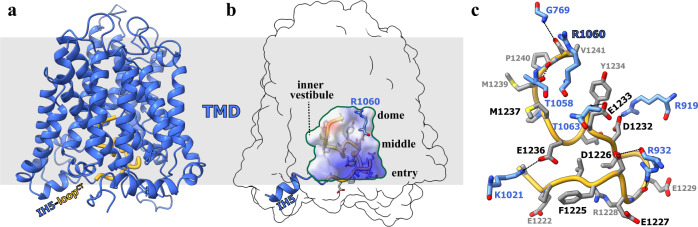
The inner vestibule and loop^CT^ in the inward-facing conformation. **a** The C-terminal loop extending from helix IH5 into the TMD helix bunding. The monomeric hAE2^acidic-KNO3^ was shown as cartoon model and colored in blue, except for the loop^CT,^ colored in yellow. **b** The inner vestibule filled with the loop^CT^. The monomeric hAE2^acidic-KNO3^ was shown as transparent surface model, while the inner vestibule was determined with Hollow and shown as the solvent-accessible electrostatic surface–potential maps. The loop^CT^ was shown as cartoon model colored in yellow and stick model colored by elements. The residue R1060 at the dome part of the inner vestibule was also shown as stick model and colored by elements. The cell membrane region was shown as gray block in (**a**) and (**b**). **c** The interactions between the loop^CT^ and the inner vestibule. The loop^CT^ was shown as cartoon model colored in yellow and stick model colored by elements (gray-blue-red), with the residues from the surface of the inner vestibule interacting with the loop^CT^ were shown as stick model colored by elements (cyan-blue-red).

All the previously reported structures of the SLC4A family transporters, AE1 (SLC4A1) and NBCE1 (SLC4A4) showed outward-facing conformation with their anion-binding pocket open to extracellular environment^27–29,34^. In the structure of hAE2^acidic-KNO3^, the acidic pH and lacking substrates keep the transporter in a resting state and adapting into an inward-facing conformation. The surface-potential analysis on the TMD shows a pyramid-shaped cytoplasmic vestibule (inner vestibule) with wide, positively charged entry near the cytoplasmic surface of the membrane, a hydrophobic middle part and a narrow dome deep into the cell membrane where a small positively charge site was identified near to the residue R1060 (Fig. 3b). This inner vestibule is surrounded by helices TM1, 5, 6, 7, 10, and 12, and could serve as the anion-binding pocket for intracellular anions loading. Interestingly, the C-terminal loop of hAE2 (loop^CT^) was found extending from the short helix IH5 into the inner vestibule and thus occupies the intracellular anion-binding pocket (Fig. 3b). The acidic residues-rich loop^CT^ is stabilized in the vestibule with the salt bridges among R919-D1232, R932-D1226, and K1021-E1236 (Fig. 3b) and the displacement of the loop^CT^ from the vestibule is necessary for anion binding and transportation.

### The conformational changes of the human AE2 upon the pH shifts

**Fig. 4:**
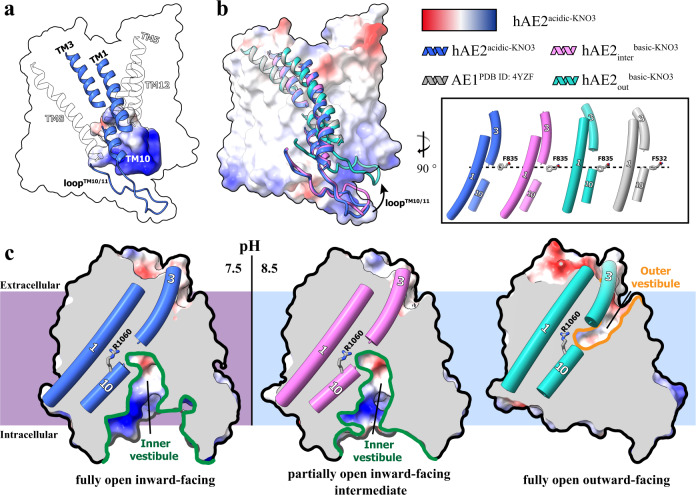
The pH shift-induced conformational changes of hAE2. **a** The helices and loops involved in conformational change. The monomeric hAE2^acidic-KNO3^ was shown as transparent surface model, while the inner vestibule was determined with Hollow and shown as the solvent-accessible electrostatic surface–potential maps. The helices surrounding the inner vestibule were shown as cartoon model, with TM1, 3, 10, and loop^TM10/11^ highlighted in blue. **b** The structural superposition among hAE2 at different pHs. The monomeric structures of hAE2^acidic-KNO3^, hAE2_inter_^basic-KNO3^, hAE2_out_^basic-KNO3^, and hAE1 were superposed, and only TM1, 3, 10, and loop^TM10/11^ were shown as cartoon model and colored in blue, pink, green, and gray, respectively. In the frame to the right was shown a side-by-side comparison among the TM1/3/10 of hAE2^acidic-KNO3^, hAE2_inter_^basic-KNO3^, hAE2_out_^basic-KNO3^, and hAE1. The residue F835 from AE2 and F532 from AE2 were shown as stick model and used as reference since their spatial locations largely remain unchanged across the models in the superposition. **c** The conformational changes and vestibule flip upon the pH shifts. The EM structures of hAE2^acidic-KNO3^ (left), hAE2_inter_^basic-KNO3^ (middle), and hAE2_out_^basic-KNO3^ (right) were shown as the solvent-accessible electrostatic surface–potential maps and sliced to display the vestibules inside the TMD. The vestibules facing the intracellular side (inner vestibule) were delineated with green lines, and the vestibules facing the extracellular side (outer vestibule) with orange line. The helices TM1, 3, and 10 were shown as cylinders cartoon model and residue R1060 as stick model colored by elements.

To further explore the conformational changes of hAE2 upon the cellular pH shift, we conducted EM studies on hAE2 at basic pHs and determined the structure of hAE2 at a pH of 8.32 (hAE2^basic-KNO3^). The 2D and 3D classification showed that the image signal for the NTD turns poor in the basic environment; thus, only the TMD of hAE2 was well solved and modeled (Supplementary Figs. 5, Fig. 8a, b). Two major classes of particles were sorted after the 3D classification, and upon the map refinement and structural modeling, two EM structures of hAE2^basic-KNO3^ were determined as homodimers in two different conformations. In the hAE2_inter_^basic-KNO3^, the hAE2 shows TM helices arrangement similar to that of hAE2^acidic-KNO3^, except that the slight tilting of helices TM1, 3, and 10 reduces the volume of the inner vestibule from 5446 to 5226 Å^3^ (Fig. 4b, c, Supplementary Fig. 11a, b), resulting in a partially open inward-facing intermediate conformation (hereinafter “intermediate”). In the other structure, hAE2_out_^basic-KNO3^, the AE2 has its TM helices arrangement apparently different from hAE2^acidic-KNO3^ but somewhat resembling hAE1 (Fig. 4b), where the inner vestibule shrinks to 1885 Å^3^, and an outer vestibule formed at the extracellular side of TMD, representing the outward-facing conformation of hAE2 (Fig. 4c and Supplementary Fig. 11c). Compared with the inner vestibule, the outer vestibule is smaller in volume (3363 Å^3^) and surrounded by helices TM1, 3, 5, and 12. Most of the surface of the outer vestibule is hydrophobic or negatively charged, except for a narrow strip of positive charges consisting of K842, K845, and R1060, probably representing the entry/exiting routine for the anion substrates (Fig. 4c and Supplementary Fig. 11d).

The co-existence of the hAE2 in the intermediate and outward-facing conformations at basic pH indicates a more flexible TM arrangement of AE2 upon the pH elevation, which might result from the disengagement of the TMD and NTD. The hAE2^acidic-KNO3^ shows that the NTD and TMD of hAE2 are locked in a stable geometry with two interactions: the hinge domain (643–672) linking the NTD with the TM1 helix of TMD, and the loop^TM10/11^ extending into and binding with NTD. Along with the elevated intracellular pH, the loop^TM10/11^ is dissociated from NTD. It lifts towards the membrane region, which loosens the interlock between TMD and NTD and allows sizeable conformational change in TMD, especially the TM helices involved with NTD-TMD interlock at acidic pH, i. e., TM1 and TM10. In a structural superposition among hAE2 in the inward-facing, intermediate, and outward-facing states, the conformational transitions were primarily driven by the displacement and tilting of TM1, 3, and 10, where all three helices move about 8–10 Å from the cytoplasmic side to the extracellular side during the inward-facing/intermediate/outward-facing transition (Supplementary Fig. 12a–c). The analysis of the various conformations of hAE2 suggests a pivotal role of R1060 in the conformational transition since it is the only positively charged residue found on the surfaces of both binding pockets at the inward-facing and outward-facing conformations. Moreover, the R1060 is located at the deepest part of both inner and outer vestibules, suggesting its role in carrying the substrate anions across the different conformations.

### The substrate-binding sites

**Fig. 5:**
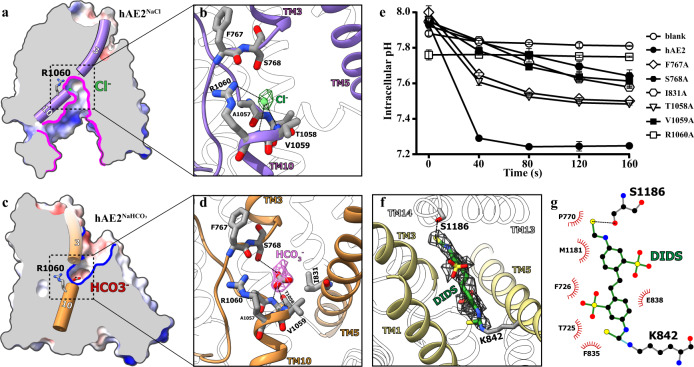
The anion substrates and inhibitor bound in the hAE2. **a** The chloride ion bound in the inner vestibule of hAE2. The hAE2^NaCl^ was shown as the solvent-accessible electrostatic surface–potential maps and sliced to show the Cl^−^ ion bound (green sphere), with the helices TM3 and 10 shown as purple cylinders and the inner vestibules delineated with pink lines. **b** The interactions among the chloride and hAE2. The dotted frame in (**a**) was enlarged, and hAE2^NaCl^ was shown as cartoon model. The Cl^−^ ion was shown as green sphere and the corresponding electron density as green mesh, with the interacting residues shown as stick model colored by elements. **c** The bicarbonate ion bound in the outer vestibule of hAE2. The hAE2^NaHCO3^ was shown as the solvent-accessible electrostatic surface–potential maps and sliced to show the HCO_3_^−^ ion bound, with the helices TM3 and 10 shown as brown cylinders and the outer vestibules delineated with blue lines. **d** The interactions among the bicarbonate and hAE2. The dotted frame in (**c**) was enlarged, and hAE2^NaHCO3^ was shown as cartoon model. The HCO_3_^−^ ion and the interacting residues shown as stick model colored by elements, with the electron density corresponding to the bicarbonate ion shown as pink mesh. **e** The cell-based function assay on the substrate-interactive mutants of hAE2. The intracellular pH changes upon the recovery of extracellular Cl^−^ were recorded for the Expi293 cells overexpressing human SLC4A2 wild-type gene and mutants as indicated to the right. The results were shown as mean ± s.d. of experiments in triplicate. **f** The interactions among the DIDS and hAE2. The hAE2^DIDS^ was shown as cartoon model and zoomed in to show the DIDS-hAE2 interactions. The TM1, TM3, and TM5 were highlighted in yellow. The DIDS and interacting residues K843 and S1185 were shown as stick model colored by elements, with the electron density corresponding to the DIDS shown as gray mesh. **g** The schematic diagram for the hAE2-DIDS interaction network as determined with LigPlot+ (v2.2.4).

To further investigate the substrate anion binding during the transportation process, EM analysis was conducted on the hAE2 protein supplemented with its natural substrate, Cl^−^ and HCO_3_^−^, as well as anion exchanger inhibitor, DIDS. In the EM maps of hAE2 with HCO_3_^−^ (hAE2^NaHCO3^) and DIDS (hAE2^DIDS^), the electron density for NTDs was not well solved, and thus only TMD structures were determined (Supplementary Fig. 8c–e). In the presence of Cl^−^ (hAE2^NaCl^), the NTDs were well determined, and the loop^TM10/11^ extends from the TMD to the NTD, similar to hAE2^acidic-KNO3^ (Supplementary Figs. 8c, 12d). On the other hand, the TM7b of hAE2^NaCl^ showed a conformational change, and its TMDs adapted into the intermediate conformation (Supplementary Fig. 12d, e). Instead of the loop^CT^-filling state of the inner vestibule of hAE2^acidic-KNO3^, the inner vestibule of hAE2^NaCl^ remains empty, and only a tiny blob of electron density was found near the residues R1060 and S768 (Fig. 5a, c and Supplementary Fig. 13a), suggesting the binding of chloride ion. The Cl^−^ ion is located near the small positively charged site on the “dome” of the inner vestibule and stabilized by the electrostatic interaction with the guanidino group of R1060 and the hydrogen bonds with backbone amides of R1060 and V1059 (Fig. 5a, b).

Both protomers of the hAE2^NaHCO3^ structure adapt into an outward-facing conformation similar to hAE2_out_^basic-KNO3^ (Supplementary Figs. 3, 12f), and similarly, a blob of non-proteinous electron density slightly larger than that identified in hAE2^NaCl^ was found in the outer vestibule near to the residues R1060 and S768 (Fig. 5c, d and Supplementary Fig. 3c), suggesting the binding of bicarbonate ion. The bicarbonate ion is stabilized by the hydrogen bonds with the hydroxyl sidechain of S768 and the backbone amides of T1058 and V1059. Additionally, the mutations on the anion-interacting residues identified in hAE2^NaCl^ and hAE2^NaHCO3^ show impaired ability in cellular pH adjustment (Fig. 5e), with their membrane expressions in comparable levels as estimated by Western blotting (Supplementary Fig. 14a, b). This further supports the two anion-binding sites identified in the inner and outer vestibules. Although the nitrate was reported as a substrate for the anion transportation by AE2^24^, the electron density was absent at the corresponding positions in the EM maps of the hAE2 in nitrate buffers free of HCO_3_^−^ (hAE2^acidic-KNO3^ and hAE2_out_^basic-KNO3^, Supplementary Fig. 13a–d). In hAE2^acidic-KNO3^, AE2 adapts to an inward-facing conformation, but the inner vestibule is occupied by the loop^CTD^, preventing anion from access to the binding site. In hAE2_out_^basic-KNO3^, a small blob of electron density could be identified near the R1060, indicating a probable binding of NO_3_^−^ with lower occupancy (Supplementary Fig. 13c, e).

DIDS is a pan-inhibitor against the anion transporters from SLC4, SLC16, and SLC26A families and chloride channels^7,35–37^. The crystal structure of AE1 (SLC4A1) bound with DIDS represents the first structural analysis of the molecular mechanism of DIDS inhibition on anion exchange, where the DIDS forms covalent bonds with K539 and K851^29^ (Supplementary Fig. 15c). Uncertainty remains about the structural basis of the inhibition by DIDS since the electron density for DIDS in the crystal structure of AE1-DIDS is not well resolved, and thus we solved the cryo-EM structure of the human AE2 supplemented with DIDS (hAE2^DIDS^). In the hAE2^DIDS^ structure, an apparent, continuous non-proteinous electron density was identified, and the whole body of the DIDS molecular fit very well (Fig. 5f and Supplementary Fig. 17). The hAE2^DIDS^ adapts into an outward-facing conformation, and the DIDS molecule lies in the middle of the outer vestibule with one of its isothiocyanato group forming the covalent bond with the ε-amino group of K842, a conserved residue corresponding to the KLIK in AE1 (Fig. 5g, Supplementary Figs. 12f, 15). On the other end of DIDS, however, the “Lys-Ser-Thr-Pro” in the TM13 of AE1 is replaced by “Met-Ser-Thr-Ala” in the TM13 of hAE2 (Fig. 5g and Supplementary Fig. 15a). As a result, the isothiocyanato group at this side shifts from TM13 to TM14, where it could be stabilized with a hydrogen bond with S1186. The cross-linking between DIDS and hAE2 tightly fixes the inhibitor molecule in the way of the substrates entering/exiting the outer vestibule and thus disrupts the pH-rebalancing capacity of AE2.

### The asymmetric intermediate state during anion exchange

In revealing the inhibitory details of the DIDS, the 2D and 3D classification indicated the existence of another class of particles without C2 symmetry. Further refinement generated an EM map with C1 symmetry at the resolution of 3.17 Å, thereby determining a TMD-only, asymmetric hAE2 dimer structure, hAE2^asymmetry^ (Supplementary Fig. 7). In this structure, two protomers adapt into two different conformations: intermediate (protomer A) and outward-facing (protomer B) (Supplementary Fig. 12g). Neither the electron densities for the DIDS in protomer B nor the loop^CT^ in protomer A is well resolved in the hAE2^asymmetry^, and thus the inner and outer vestibules remain largely empty (Supplementary Fig. 8f). This hybrid dimer structure shows that the two exchanger subunits in the dimeric AE2 might not function synchronously, and the relatively more minor conformational change in the helices of gate domains makes them form a relatively rigid interface to maintain the dimeric architecture during the anion exchange, thereby allowing the two flexible core domains of dimer operate independently. This asymmetric conformation is not limited to the AE2 supplemented with DIDS, and a 3D-class in the asymmetric conformation could also be identified from the hAE2 sample unsupplemented with DIDS (hAE2_NaHCO3_^asymmetry^). Further refinement on this class resulted in an EM map of resolution lower than 4.6 Å (Supplementary Fig. 4), and its overall conformation for the main chain is the same as the one with DIDS (Supplementary Fig. 16). This suggests that the asymmetry conformation is independent of inhibitors, and thereby no molecular model was built in this map of lower quality.

### The mechanistic implication for the pH shift-induced anion exchange

**Fig. 6:**
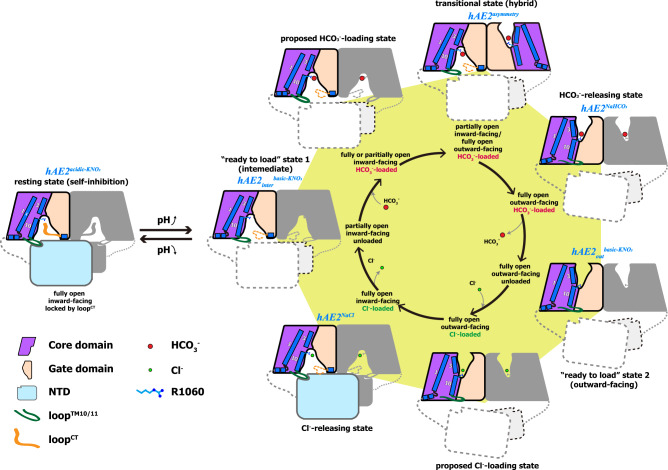
The working model for the pH-mediated anion exchange by human AE2. The hAE2 stays in the resting state (left) at physiological intracellular pH and will enter the operating cycle (shown as the heptagon to the right) upon the pH elevation. The EM structures representing the working states of the hAE2 were labeled above the corresponding cartoon model in italics in blue. The conformations and anions bound for each working state are shown in the inner cycle.

Our EM structures of human AE2 show its multiple operating states in the anion-exchanging process and the triggering conditions in the transitions among the different states (Supplementary Movie 1). As shown in Fig. 6, the AE2 stays in a self-inhibition resting state at physiological pH where the loop^CT^ occupies the inner vestibule to block the substrate binding and the loop^TM10/11^ inserts into NTD to interlock the TMD helices, as shown by hAE2^acidic-KNO3^. When the cellular pH raises along with the acid secretion, the pH shift-induced conformational change, possibly in NTDs, would release the loop^TM10/11^ from NTD to allow the helical re-arrangement in TMD, resulting in the removal of loop^CT^ from the inner vestibule and thus allowing the access of bicarbonates to the substrate binding site (“ready to load” state, hAE2_inter_^basic-KNO3^). Our EM analysis shows a predominant outward-facing conformation when hAE2 was supplemented with NaHCO_3_, suggesting the loading of the bicarbonates on the substrate binding site could induce a dramatic flip of the TM helices bundle and switch the inward-facing/HCO_3_^−^-bound conformation (proposed HCO_3_^−^-loading state) into the outward-facing/HCO_3_^−^-bound conformation (“HCO_3_^−^-releasing” state, hAE2^NaHCO3^). Similarly, the loading of extracellular Cl^−^ onto the substrate binding site in the outer vestibule would drive the switch from the outward-facing/Cl^−^-bound conformation to the inward-facing/Cl^−^-bound conformation as suggested by the predominance of hAE2 in inward-facing conformation when supplemented with NaCl (“Cl^−^-releasing” state, hAE2^NaCl^), thereby releasing the Cl^−^ into the cytoplasm to complete the anion exchange cycle. The AE2 could stay in the operating cycle for a continuous Cl^−^ influx/HCO_3_^−^ efflux until the cellular pH reduces to the physiological value and AE2 returns to its self-inhibition state. In addition to the aforementioned major operating states, the hAE2 in two different conformations were found to co-exist as a hybrid dimer (hAE2^asymmetry^), indicating a transitional state during the inward-/outward-facing conformational switch.

### The structural comparison among SLC4A family

Previous structural studies on the human SLC4 family have presented several structures for AE1 (SLC4A1)^27–29^, NBCE1 (SLC4A4)^34^, and NDCBE (SLC4A8)^38^. Those structures provided deep insights into the transmembrane helices’ organization in outward-facing conformations and the substrate-binding sites in the outer vestibule. At the same time, the information about the transportation and regulatory mechanisms was limited since the entire architecture of NTD-TMD and their inward-facing conformation still need to be clarified (Supplementary Fig. 18a). In a structural superposition among hAE1 structures versus hAE2 of inward-facing (Supplementary Fig. 18b) or outward-facing conformation (Supplementary Fig. 18c), the hAE2 in the outward-facing conformation shows high structural similarities with the hAE1s, including a crystal structure of TM-only AE1 and two cryo-EM structures of full-length AE1 in complex with band 4.2 protein^28^ and glycophorin-A^27^. Meanwhile, the comparison among the hAE2 in the inward-facing conformation and hAE1s shows significant differences in their TMs organization (especially in the core region) and loop^TM10/11^, with overall RMSDs ranging from 5.2 to 5.5 Å. Although all determined at physiological conditions, human SLC4A1, 4, and 8 show outward-facing conformations, while AE2 adapts to an inward-facing conformation at pHs lower than 7.25 and outward-facing conformation at a pH as high as 8.32, indicating its different pH-response comparing with other three SLC4 members. In light of the self-inhibition resting state found in the inward-facing conformation of AE2, we assume that AE2 tends to stay inactive at physiological pH since its major workplace, acid-secreting cells such as gastric cells and osteoclasts, need the pH-lowering activities of AE2 only upon the cellular alkalization.

## Discussion

Cells need to maintain strict pH homeostasis for the biological functions of macromolecules. The pH might shift following metabolic reactions or specific cellular functions such as acid secretion, entailing a pH-sensitive pH adjustment system. In this study, we combine structural biology and cell-based functional assay to demonstrate the structural basis for the pH-sensitive anion exchange catalyzed by human AE2. AE2 has been established as a pH-regulator in acid-producing cells such as the osteoclasts and gastric parietal cells, where it catalyzes exchange between the intracellular bicarbonate and extracellular chloride across the basal membrane. The four EM structures determined here represent the four essential working states of AE2: the resting state (self-inhibitory fully open inward-facing intermediate conformation), the ready-to-load state (partially open inward-facing intermediate conformation), the bicarbonate-releasing state (fully open outward-facing HCO_3_^−^ loaded conformation), the chloride-releasing state (fully open inward-facing Cl^−^ loaded conformation). The structure of AE2 bound with HCO_3_^−^ can only be solved in an outward-facing conformation, suggesting the binding of HCO_3_^−^ could induce a quick switch between inward and outward-facing conformation, and an inward-facing HCO_3_^−^ bound conformation could be a transient state. The same logic applies to the chloride binding, which results in a conformational switch in the opposite direction.

The HCO_3_^−^ binding might not be the only triggering factor for the switch between the inward- and outward-facing conformations since the hAE2_out_^basic-KNO3^ structure show that part of HCO_3_^−^-deprived hAE2 can also adapt into outward-facing conformation at basic pH and thus a mixture of inward-facing dimers and outward-facing dimers was formed in the absence of HCO_3_^−^ upon the pH elevation. Furthermore, with the assistance of DIDS, a hybrid dimeric conformation could be identified with both conformations in the same dimeric AE2, which shows the functional independence of the protomers from the AE2 dimer and might represent a transitional state during the conformational switch. Those subtle conformational variations reveal the structural details in the dynamic process of anion exchange and show that an adaptive regulatory mechanism might exist, allowing the AE2 to work at the basic pH even if one of its substrates is running low.

Although the elevated pH was considered as a triggering condition for hAE2-mediated anion transportation, and our EM structures clearly show a conformational change upon the pH shift, the pH-sensing mechanism of AE2 remains ambiguous. One of the significant difficulties is the structural instability of AE2 at basic pHs. Our EM analysis showed that the electron density for the AE2 NTD could be well solved at physiological pH or below. It will turn discontinuous and poor at a pH of 8.5 or higher, ending with TMD-only structures without NTD. Nevertheless, we could still reasonably speculate on the pH-sensor function of the AE2 NTD since the worsening of the density of AE2 NTD upon the pH elevation indicates significant conformational change occurs at NTD in response to the pH elevation. Another structural implication was presented by the “escapement” mechanism of the loop^TM10/11^-NTD interaction. As shown in Fig. 2a, c, and Supplementary Fig. 4e, this interaction is only engaged at the physiological pH and results in an interlocked state, preventing the conformation change in the TMD. Along with the rising pH, the interaction will be disrupted by the conformational change in NTD, and thus the loop^TM10/11^ is released from NTD to allow the rearrangements of the TMD helices.

Based on the six EM structures of human AE2 in different conformations/states, further questions about the anion exchange emerge to be addressed. Future efforts for the pH-induced conformational change in NTD are warranted for its importance in elaborating the detailed starting mechanism of the anion exchange activity of AE2. Besides, although we proposed the self-inhibition of the AE activities by the loop^CT^-occupation of the inner vestibule, further functional studies upon the mutations on the loop^CT^-inner vestibule interface should be performed to confirm this self-inhibition effect and the detailed regulatory mechanism is worthy of further investigation to understand the process of retrieving the loop^CT^ from the inner vestibule and the way AE2 prevents the unnecessary re-binding of loopCT in its operating cycle.

In conclusion, given the presently revealed structural models, the AE2 uses NTD as the intracellular pH sensor to mediate the anion-exchanging activities of its TMD, thereby maintaining pH homeostasis.

## Methods

### Expression constructs

The different isoforms of human SLC4A2 gene cDNAs were acquired from Han Jiahuai lab at Xiamen University and then cloned into the mammalian cells expression vector pCAGGS (Addgene) modified to introduce a Flag tag plus an 8 × His tag followed by a Tobacco Etch Virus (TEV) protease cleavage site at the N-terminal of AE2. The Quick-change site-directed mutagenesis method was used to introduce the mutations. All the mutants and wide-type constructs were confirmed by DNA sequencing before functional and structural studies.

### Protein expression and purification

The suspension-cultured Expi293 cells (Thermofisher, A14528) were used for protein expression. The SLC4A2c encoding the 177 to 1241 aa of human AE2 canonical sequence (UniProt ID P04920) showed high yield and stability as estimated by the size-exclusion chromatography and negative staining electron microscopy and thus was chosen for further functional and structural studies. Generally, when cell density reached about 2.5 × 10^6^ cells mL^−1^, plasmids carrying expression constructs for hAE2 or mutants were transiently transfected into the cells using PEI MAX (Polysciences). For structural studies, the transfected cells were further cultured for 72 h and harvested by centrifugation at 1500 *g* for 10 min. Cell pellets can freeze at −80 °C until further use.

Protein purifications were carried out at 4 °C or ice bathed. Firstly, cell pellets were resuspended and homogenized in a Low Salt buffer (10 mM KNO_3_, 10 mM HEPES, pH 7.25) containing 1 mM PMSF (Phenylmethanesulfonyl fluoride), and the cell debris containing target protein was harvested by centrifugation (45,000 *g*, 25 min, 4 °C). Then the pellets were resuspended and homogenized in a High Salt buffer (1 M KNO_3_, 25 mM HEPES, pH 7.25) containing 1 mM PMSF, 5 mM Mg(NO_3_)_2_, 0.1 mg mL^−1^ DNase I and centrifugated again to collect the membrane debris. Finally, the pellets were resuspended and homogenized in Lysis Buffer (150 mM KNO_3_, 20 mM HEPES, pH 7.25, 10% (v/v) Glycerol) in the presence of 5 mM Mg(NO_3_)_2_, 0.1 mg mL^−1^ DNase I, 1 × protease inhibitor cocktail (MCE) and the membrane was solubilized by adding 1% (w/v) lauryl maltose neopentyl glycol (LMNG, Anatrace), 0.1% (w/v) cholesterol hemisuccinate (CHS, Anatrace), 0.01% (w/v) glyco-diosgenin (GDN, Anatrace) and gently stirring at 4 °C for 2 h. After centrifugation at 45,000 g for 45 min at 4 °C, the supernatant was subjected to affinity chromatography using anti-Flag beads (GeneScript), and the proteins were eluted using the Lysis Buffer supplemented with 200 μg mL^−1^ 3×flag peptide and 0.01 % (w/v) glyco-diosgenin (GDN, Anatrace). The extracted proteins were further separated by size-exclusion chromatography using Superose 6 Increase 10/300 GL column (Cytiva) in a mobile phase containing 150 mM KNO_3_, 20 mM HEPES, pH 7.25, and 0.01% (w/v) GDN. The dimer fractions were pooled and concentrated to about 10 mg mL^−1^ for cryo-EM grids preparation. When prepared hAE2 in the presence of Cl^-^, NaCl, and MgCl_2_ was used to replace the KNO_3_ and Mg(NO_3_)_2_ during the purification. When prepared hAE2 with NaHCO_3_ and in basic conditions, 50 mM NaHCO_3_ (Aladdin) and 100 mM Tris-HCl (pH 8.32) were added and incubated for 30 min before cryo-grids preparation, respectively. The DIDS-bounded sample was prepared by adding 0.5 mg mL^−1^ DIDS (Sigma) and 50 mM NaHCO_3_.

### Cryo-EM sample preparation, data collection, and processing

The cryo-grids were prepared using Thermo Fisher Vitrobot Mark IV operated at 8 °C with 100% humidity. About 3 μL (~10 mg mL^−1^) of hAE2 in different conditions were applied to glow-discharged holey carbon grids (Quantifoil R1.2/1.3, Au, 300 mesh). The protein sample was incubated for 10 s and then blotted with filter paper (Waterman) for 1.5 s. The grids were then plunged into the liquid ethane and cooled with liquid nitrogen. The cryo-grids were loaded on 200 kV cryo-EM (FEI, Talos Arctica) to check protein concentration and the quality of particles. The high-resolution images were collected on a 300 kV cryo-EM (FEI, Titan Krios) equipped with a K3 Summit direct electron detector (Gatan), a Quantum energy filter (Gatan), and a Cs corrector (Thermo Fisher), functioning in zero-energy-loss mode with the slit width of 15 eV. The movie stacks were automatically collected using EPU software at a nominal magnification of 81,000 × (corresponding to a physical pixel size of 1.1 Å), with a defocus range between −1.0 and −2.5 μm. The dose rate was set to ~13.9 e^−^/Å^2^/s, and the total exposure time was 4.32 s, resulting in a total dose of 50 e^−^/Å^2^, fractionated into 32 frames.

All the cryo-EM data processing was performed using programs RELION-3.1.1 and cryoSPARC-v3.2.0^32,39^. In summary, five datasets were collected, and the overall information is listed in Supplementary Table 1. The motion correction was performed using MotionCorr2 with 6 × 5 patches^40^, and the CTF parameters were estimated using Gctf with the images of poor statistics being discarded^41^. Particles were automatically picked using Gautomatch-v0.56 (developed by Kai Zhang), and a small fraction of particles after 2D classification were used to generate initial references using ‘Ab-Initio Reconstruction’ in cryoSPARC. For each full dataset, the poor particles were excluded through one cycle 2D classification at 4×binning, following multi-iterations of 3D classification at 2 × binning through the Relion-3 or cryoSPARC, and particles in the best class were re-extracted in 1 × binning pixel size followed by Bayesian polishing in RELION-3. The polished particles were re-subjected to cryoSPARC, then Non-uniform Refinement with C2 symmetry was performed, thereby yielding a high-resolution map. Local resolution estimation was performed in cryoSPARC (Local Resolution Estimation), and all the resolutions were estimated using the gold-standard Fourier shell correlation 0.143 criteria with the high-resolution noise substitution. All the detailed data information and processing procedures were illustrated in Supplementary Table 1 and Supplementary Figs. 9–13.

### The intracellular pH measurement

The intracellular pH measurement was performed using the intracellular pH indicator BCECF-AM. BCECF-AM is colorless and can freely permeate cell membranes, followed by the isomerases-hydrolysis to generate fluorescent BCECF. The fluorescence excitation profile of BCECF is pH-dependent. The biggest excitation wavelength of BCECF is 488 nm, and the ratio of emission fluorescence intensities measured at 535 nm (E_m_535) and 661 nm (E_m_661) can be used to calculate the intracellular pH^42^. The Expi293 cells were used for functional studies, and the Intracellular pH Calibration Buffer Kit (Thermo) was used to generate the pH standard curve for intracellular calculation, following the formula y = 1.4107x + 4.1808, where the y represents the intracellular pH, and the x represents the ratio of E_m_535/E_m_661. All assays were performed in triplicate in at least three separate experiments. The hAE2 wild type and mutants of interest were transfected into Expi293 cells, and each group of cells (for each AE2 mutant or wild type) was split into three vials for separate measurements.

### The anion exchange activity determination for hAE2

For anion exchange activity measurement, the Expi293 cells transfected with the expression vectors carrying hAE2 and different mutants were cultured for 48 h and incubated with BCECF-AM (Sigma) at the concentration of 1 μg mL^−1^ in an FBS-free medium at 37 °C for 30 min. The extra fluorescent dye was removed by washing the cells in an FBS-free medium twice. Then the cells were resuspended in Cl^-^-Free Buffer (140 mM Na-Gluconate, 5 mM K-Gluconate, 1 mM MgSO_4_, 1 mM Ca-Gluconate, 5 mM Glucose, 25 mM NaHCO3, 10 mM HEPES, 2.5 mM NaH_2_PO_4_, pH 7.4) and incubated at room temperature for 10 min. Aliquots of cells were subjected to flow cytometry to measure the emission fluorescence intensities at 530 nm and 661 nm (BD LSRFortessa). The remaining cells were centrifugated and transiently resuspended with a Cl^-^-Buffer (140 mM NaCl, 5 mM KCl, 1 mM MgSO_4_, 1 mM Ca-Gluconate, 5 mM Glucose, 25 mM NaHCO_3_, 10 mM HEPES, 2.5 mM NaH_2_PO_4_, pH 7.4) and the emission fluorescent intensities at 530 nm and 661 nm were recorded every 40 s. The intracellular pH was calculated using the standard curve, and the pH-time curves were generated using GraphPad Prism.

### Model building and refinement

The structural modeling for hAE2^acidic-KNO3^ was performed with the program Coot by merging the transmembrane domain structure of AE1 (4YZF, [10.2210/pdb4YZF/pdb]) and the NTD structure of AE1 (1HYN, [10.2210/pdb1HYN/pdb]) as the initial model, and the initial model was iteratively adjusted and refined with the Real-space refinement in Phenix package and Coot^43,44^. All the other hAE2 models were also built in Coot using the structure of hAE2^acidic-KNO3^ as the starting model, followed by several iterations’ refinement in Phenix and Coot. The DIDS, HCO_3_^−^ and Cl^−^ molecules were fit and refined using the program LigandFit and Real-space refinement in the Phenix package, respectively.

### Western blot

The same batches of cells for functional studies were collected and resuspended in Lysis Buffer (150 mM NaCl, 20 mM HEPES, pH 7.25) in the presence of 5 mM MgCl_2_, 0.1 mg mL^−1^ DNase I, 1 × protease inhibitor cocktail. DDM was added to solubilize the membrane proteins at 4 °C for 2 h. The solubilization slurries were clarified by centrifugation, and the supernatant was subjected to SDS-PAGE followed by Western blot. We used the Mouse anti DDDDK-Tag mAb (ABclonal, AE005) to detect the expression of hAE2 and different mutants. The secondary antibody is Anti-mouse IgG, HRP-linked Antibody (Cell Signaling, #7076). We also use the GAPDH Rabbit mAb (Abclonal, Catalog number: A19056) to evaluate the cell quantity. The secondary antibody is Anti-rabbit IgG, HRP-linked Antibody (transgen, Catalog number: HS101-01). All the antibodies were diluted (1:10,000) with skim milk in TBST in prior to use. Images were captured with an Amersham Imager 600.

### Reporting summary

Further information on research design is available in the Nature Portfolio Reporting Summary linked to this article.

## Supplementary information

Supplementary Information

Peer Review File

Description of Additional Supplementary Files

Supplementary Movie 1

Reporting Summary

## Data Availability

The data that support this study are available from the corresponding authors upon request. The cryo-EM maps have been deposited in the Electron Microscopy Data Bank (EMDB) with accession codes EMDB-34293 (AE2^acidic-KNO3^), EMDB-34292 (AE2_out_^basic-KNO3^), EMDB-34289 (AE2_inter_^basic-KNO3^), EMDB-34288 (AE2^NaCl^), EMDB-34290 (AE2^NaHCO3^), EMDB-34287 (AE2^DIDS^), EMDB-34291 (AE2^asymmetry^). The coordinates have been deposited in the Protein Data Bank PDB with the accession codes 8GVH (AE2^acidic-KNO3^), 8GVF (AE2_out_^basic-KNO3^), 8GVA (AE2_inter_^basic-KNO3^), 8GV9 (AE2^NaCl^), 8GVC (AE2^NaHCO3^), 8GV8 (AE2^DIDS^), and 8GVE (AE2^asymmetry^).  Source data are provided with this paper.

## Source data

Source Data
