# Supplementary material for: The structural basis of the pH-homeostasis mediated by the Cl−/HCO3− exchanger, AE2
Source: Nat Commun. 2023 Mar 31;14:1812. doi: 10.1038/s41467-023-37557-y (PMC10066210; doi:10.1038/s41467-023-37557-y)
Supplement: Supplementary file 1 — Supplementary Information [file 41467_2023_37557_MOESM1_ESM.pdf]

Supplementary Information for

The structural basis of the pH-homeostasis mediated by the Cl^-/HCO_3^- exchanger, AE2

Qing Zhang[1,2†], Liyan Jian[1,3†], Deqiang Yao[2,4†], Bing Rao[1,2], Ying Xia[2], Kexin Hu[2], Shaobai Li[2], Yafeng Shen[2], Mi Cao[2], An Qin[1,3], Jie Zhao[1,3], Yu Cao[1,2*]

Supplementary text includes:
Supplementary Figs. 1 to 18
Supplementary Tables 1 to 2-2
Movie S1

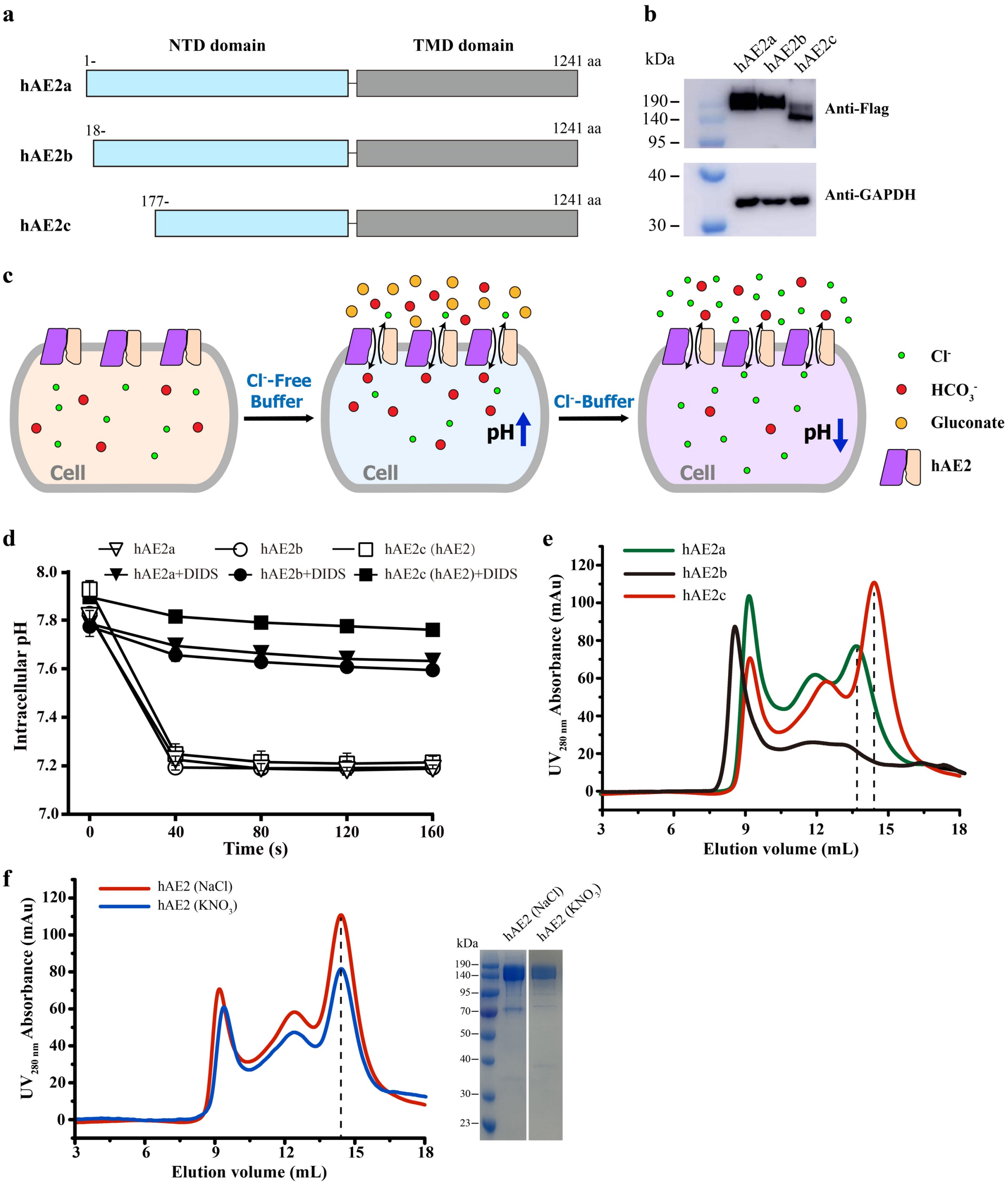

Supplementary Fig. 1. The anion exchange activity measurement and the sample preparation of hAE2. **(a)** Three isoforms of human AE2. The isoform hAE2a is encoded by the human SLC4A2 gene consisting of residues 1-1241 (UniProt ID P04920), with residues 18-1241 for hAE2b and 177-1241 for hAE2c. **(b)** The western blot analysis of the recombinant expression levels of hAE2 of three isoforms. The expression levels of hAE2a, hAE2b, and hAE2c (hAE2, 177-1241 aa) were detected using an anti-Flag antibody. The result shown is representative of three replicates. **(c)** The experimental principle for the cell-based assay for the anion exchange activity of hAE2. **(d)** The cell-based activity assay of the human AE2 isoforms. The intracellular pH changes upon the recovery of extracellular Cl^- were recorded for the Expi293 cells overexpressing human hAE2a, hAE2b, and hAE2c in the presence or absence of DIDS inhibitor. The results were shown as mean ± s. d. of experiments in triplicate. **(e)** The size exclusion chromatography profiles of hAE2 in different isoforms. The dashed line indicates the fractions used for cryo-EM sample preparation. **(f)**

The purification of human AE2. The FPLC profiles show the purified hAE2 proteins in different buffers, and the SDS-PAGE analysis shows the eluates from the affinity chromatography in different buffers. The dashed line indicates the fractions used for cryo-EM sample preparation. The size exclusion has been repeated more than three times with similar results.

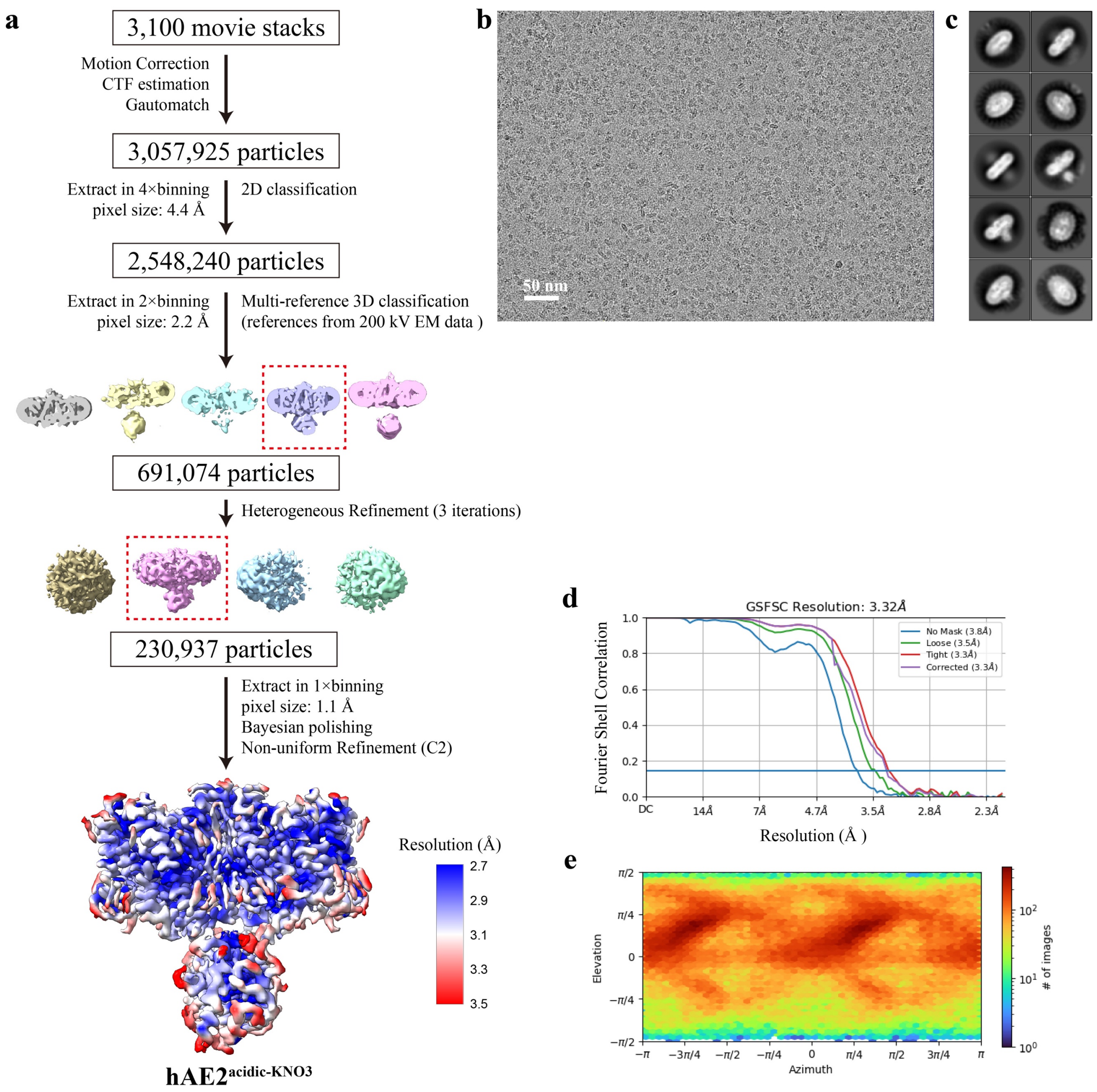

Supplementary Fig. 2. The cryo-EM analysis of hAE2$^{acidic\text{-}KNO3}$. (a) The flow chart of cryo-EM data processing on hAE2$^{acidic\text{-}KNO3}$. Local-resolution map shown in blue-white-red. **(b)** A representative cryo-EM micrograph of hAE2$^{acidic\text{-}KNO3}$. Most of the micrographs were similar with high quality. **(c)** A representative 2D class averages. **(d)** The gold-standard Fourier shell correlation (FSC) curve for the final cryo-EM map of hAE2$^{acidic\text{-}KNO3}$, generated by cryoSPARC with non-uniform refinement. **(e)** Orientation distribution of particles for the final map reconstruction.

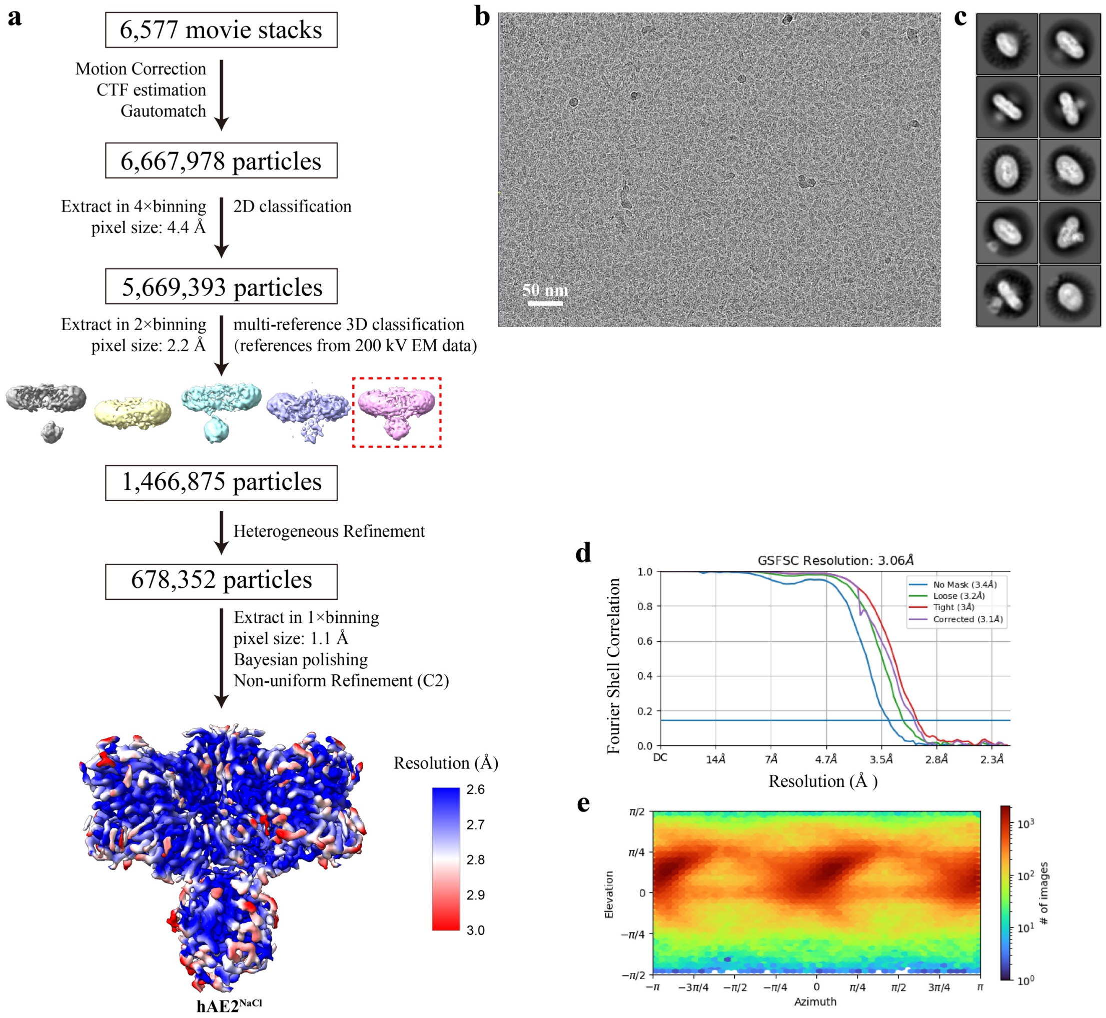

Supplementary Fig. 3. The cryo-EM analysis of hAE2^{NaCl}. (a) The flow chart of cryo-EM data processing on hAE2^{NaCl}. Local-resolution map shown in blue-white-red. **(b)** A representative cryo-EM micrograph of hAE2^{NaCl}. Most of the micrographs were similar with high quality. **(c)** A representative 2D class averages. **(d)** The gold-standard Fourier shell correlation (FSC) curve for the final cryo-EM map of hAE2^{NaCl}, generated by cryoSPARC with non-uniform refinement. **(e)** Orientation distribution of particles for the final map reconstruction.

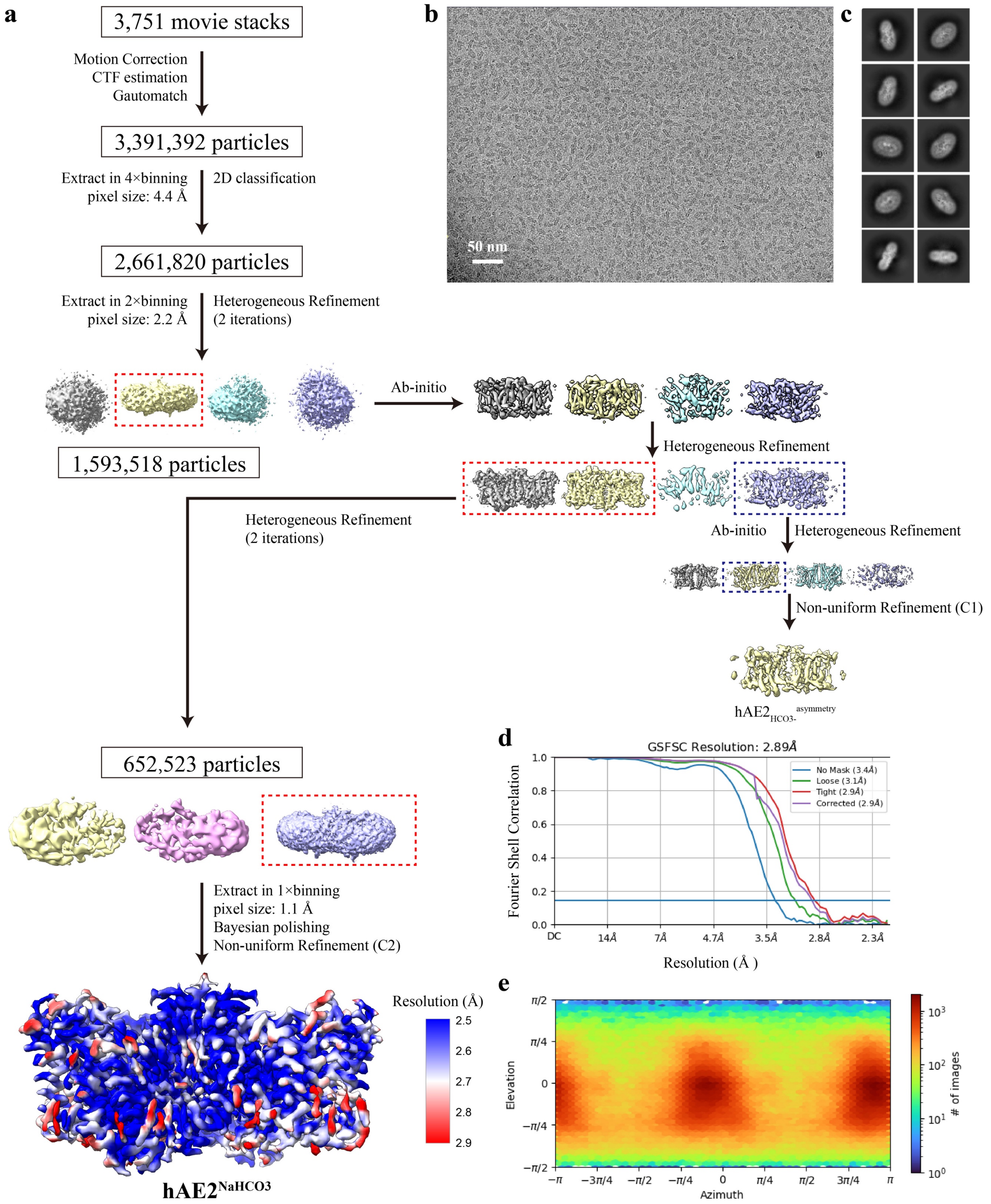

Supplementary Fig. 4. The cryo-EM analysis of hAE2^{NaHCO3}. (a) The flow chart of cryo-EM data processing on hAE2^{NaHCO3}. Local-resolution map shown in blue-white-red. **(b)** A representative cryo-EM micrograph of hAE2^{NaHCO3}. Most of the micrographs were similar with high quality. **(c)** A representative 2D class averages. **(d)** The gold-standard Fourier shell correlation (FSC) curve for the final cryo-EM map of hAE2^{NaHCO3}, generated by cryoSPARC with non-uniform refinement. **(e)** Orientation distribution of particles for the final map reconstruction.

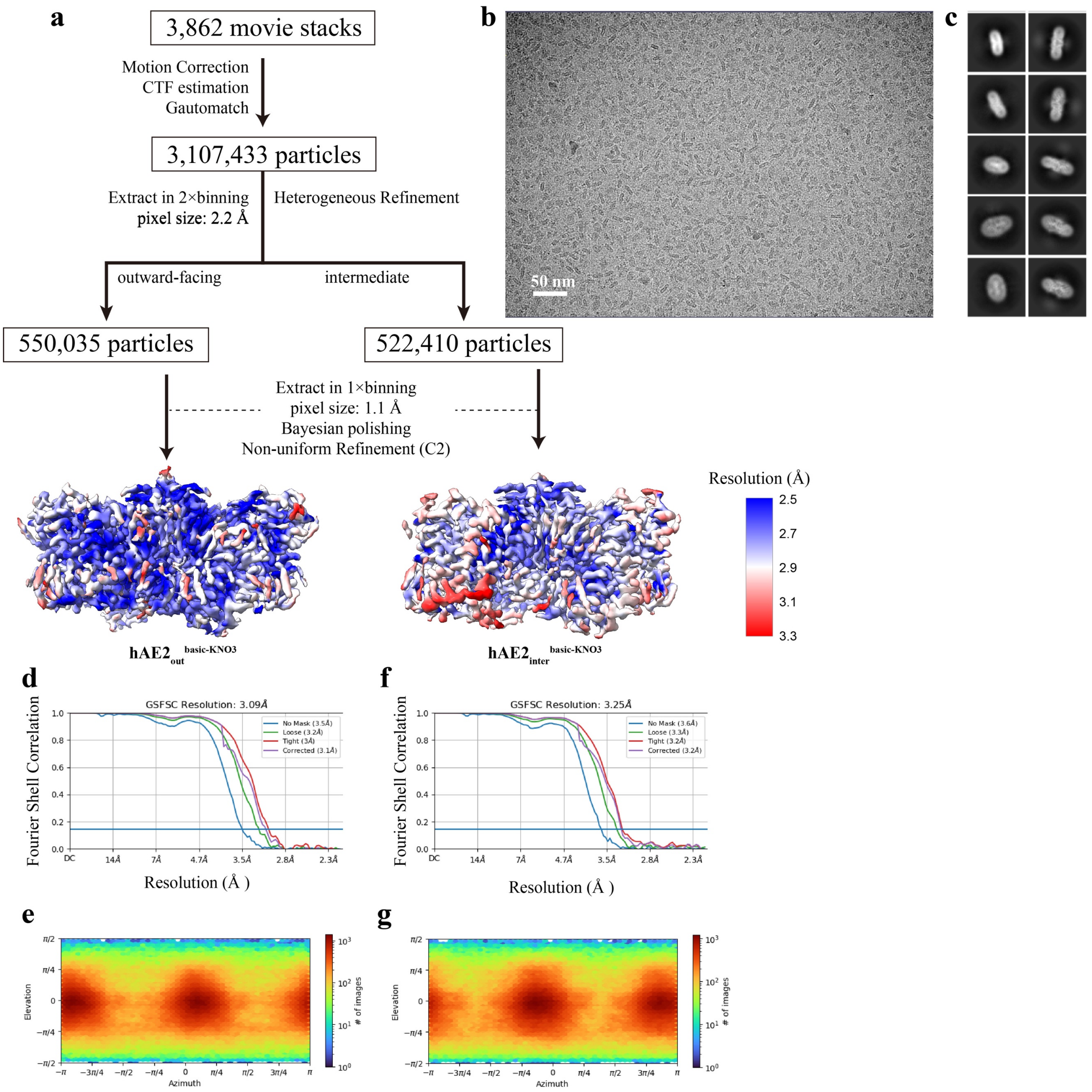

Supplementary Fig. 5. The cryo-EM analysis of AE2$^{basic-KNO3}$. (a) The flow chart of cryo-EM data processing on hAE2$^{basic-KNO3}$. The Local-resolution maps for AE2$_{inter}^{basic-KNO3}$ and AE2$_{out}^{basic-KNO3}$ were shown in blue-white-red, respectively. **(b)** A representative cryo-EM micrograph of hAE2$^{basic-KNO3}$. Most of the micrographs were similar with high quality. **(c)** A representative 2D class averages. **(d)** The gold-standard Fourier shell correlation (FSC) curve for the final cryo-EM map of AE2$_{inter}^{basic-KNO3}$, generated by cryoSPARC with non-uniform refinement. **(e)** Orientation distribution of particles for the final map reconstruction of AE2$_{inter}^{basic-KNO3}$. **(f)** The gold-standard Fourier shell correlation (FSC) curve for the final cryo-EM map of AE2$_{out}^{basic-KNO3}$, generated by cryoSPARC with non-uniform refinement. **(g)** Orientation distribution of particles for the final map reconstruction of AE2$_{out}^{basic-KNO3}$.

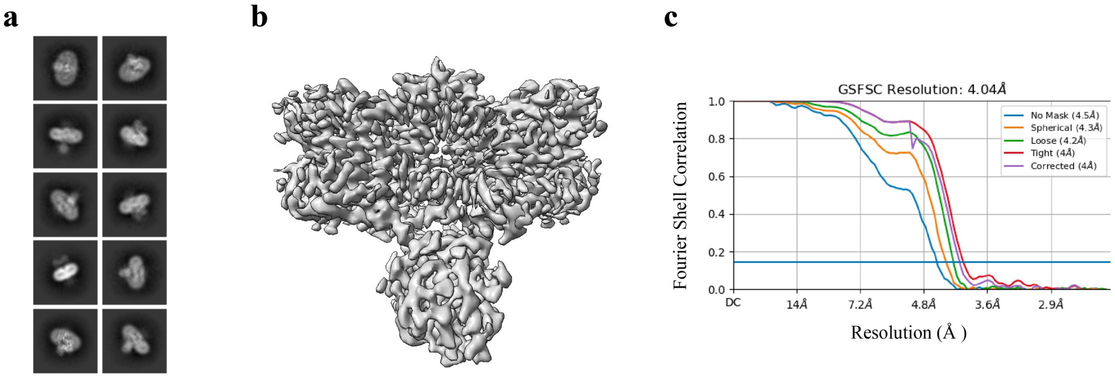

Supplementary Fig. 6. The cryo-EM analysis of hAE2 at pH 6.55. (a) A representative 2D class averages. **(b)** The cryo-EM map of hAE2 at pH 6.55 as viewed parallel to the cell membrane. **(c)** The gold-standard Fourier shell correlation (FSC) curve for the final cryo-EM map. The overall resolution is 4.04 Å.

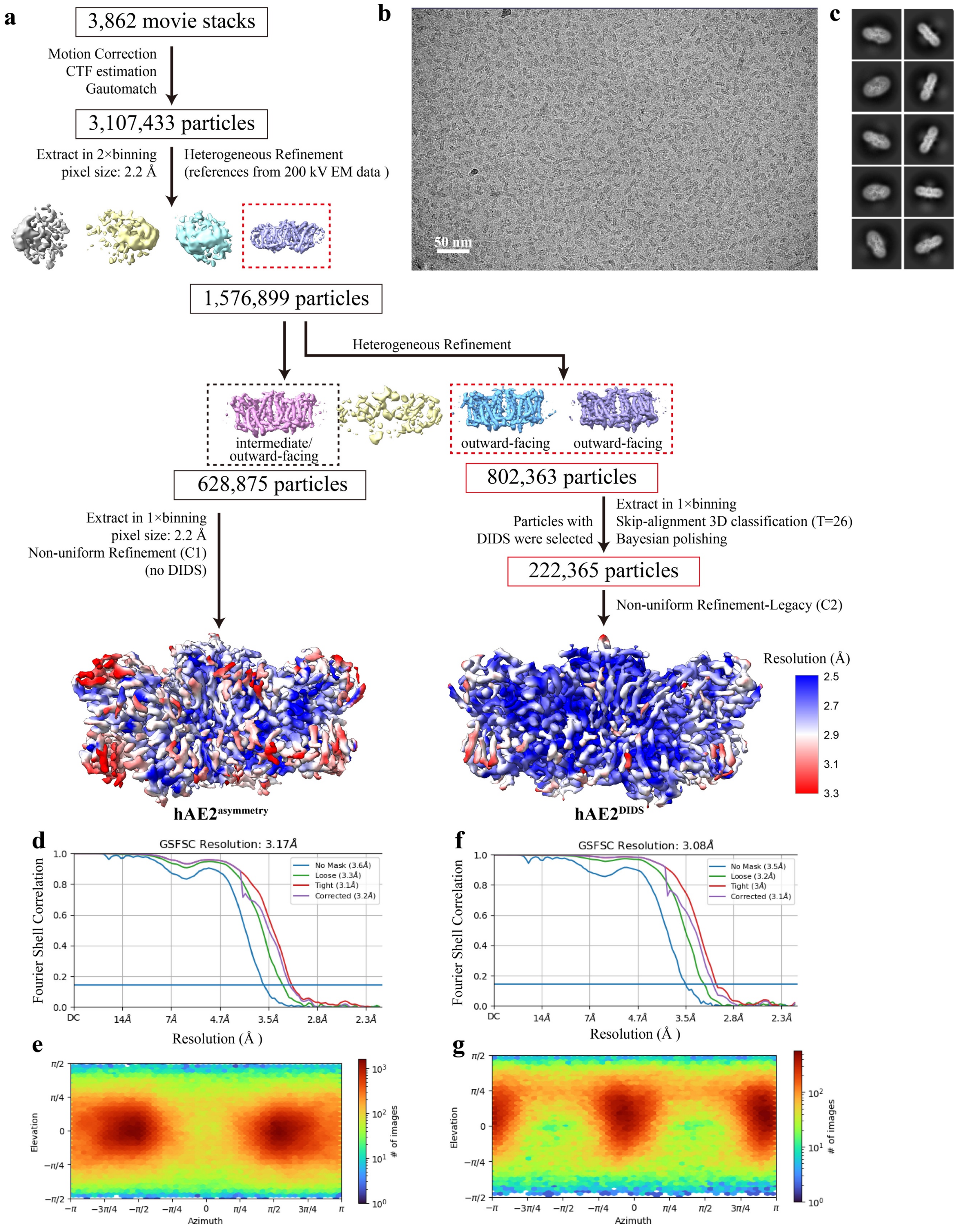

Supplementary Fig. 7. The cryo-EM analysis of hAE2 with DIDS. (a) The flow chart of cryo-EM data processing. on hAE2^{DIDS}. The Local-resolution maps for AE2^{asymmetry} and AE2^{DIDS} were shown in blue-white-red, respectively. **(b)** A representative cryo-EM micrograph of hAE2 with DIDS. Most of the micrographs were similar with high quality. **(c)** A representative 2D class averages. **(d)** The gold-standard Fourier shell correlation (FSC) curve for the final cryo-EM map of AE2^{asymmetry}, generated by cryoSPARC with non-uniform refinement. **(e)** Orientation distribution of particles for the final map reconstruction of AE2^{asymmetry}. **(f)** The gold-standard Fourier shell correlation (FSC) curve for the final cryo-EM map of AE2^{DIDS}, generated by

cryoSPARC with non-uniform refinement. **(g)** Orientation distribution of particles for the final map reconstruction of AE2^{DIDS}.

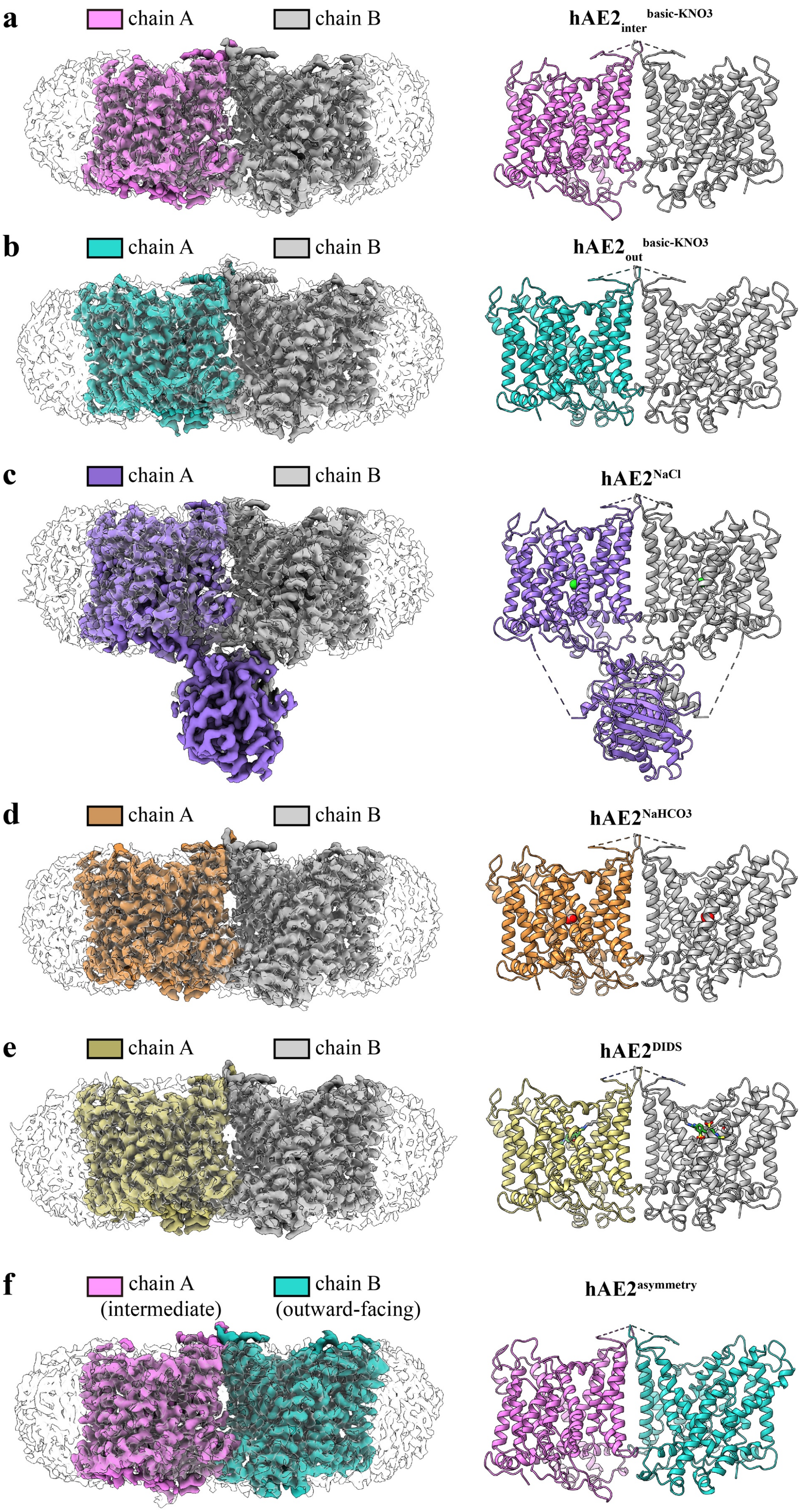
a
chain A
chain B
hAE2inter basic-KNO3
b
chain A
chain B
hAE2out basic-KNO3
c
chain A
chain B
hAE2NaCl
d
chain A
chain B
hAE2NaHCO3
e
chain A
chain B
hAE2DIDS
f
chain A
(intermediate)
chain B
(outward-facing)
hAE2asymmetry

Supplementary Fig. 8. The cryo-EM density maps and the corresponding models of hAE2 in different states. **(a)** The density map (left) and the cartoon model of $hAE2_{inter}^{basic-KNO3}$. Two protomers were colored in pink and grey, respectively. **(b)** The density map and the cartoon model of $hAE2_{out}^{basic-KNO3}$. Two protomers were colored in green and grey, respectively. **(c)** The density map and the cartoon model of $hAE2^{NaCl}$. The Cl^- ions were shown as green sphere, and two protomers of hAE2 were colored in purple and grey, respectively. **(d)** The density map and the cartoon model of $hAE2^{NaHCO3}$. The HCO_3^- ions were shown as stick model colored by elements, and two protomers of hAE2 were colored in orange and grey, respectively. **(e)** The density map and the cartoon model of $hAE2^{DIDS}$. The DIDS was shown as stick model in green, and two protomers of hAE2 were colored in yellow and grey, respectively. **(f)** The density map and the cartoon model of $hAE2^{asymmetry}$. The protomer in the intermediate state was colored in pink, and the protomer in the outward-facing state was colored in green.

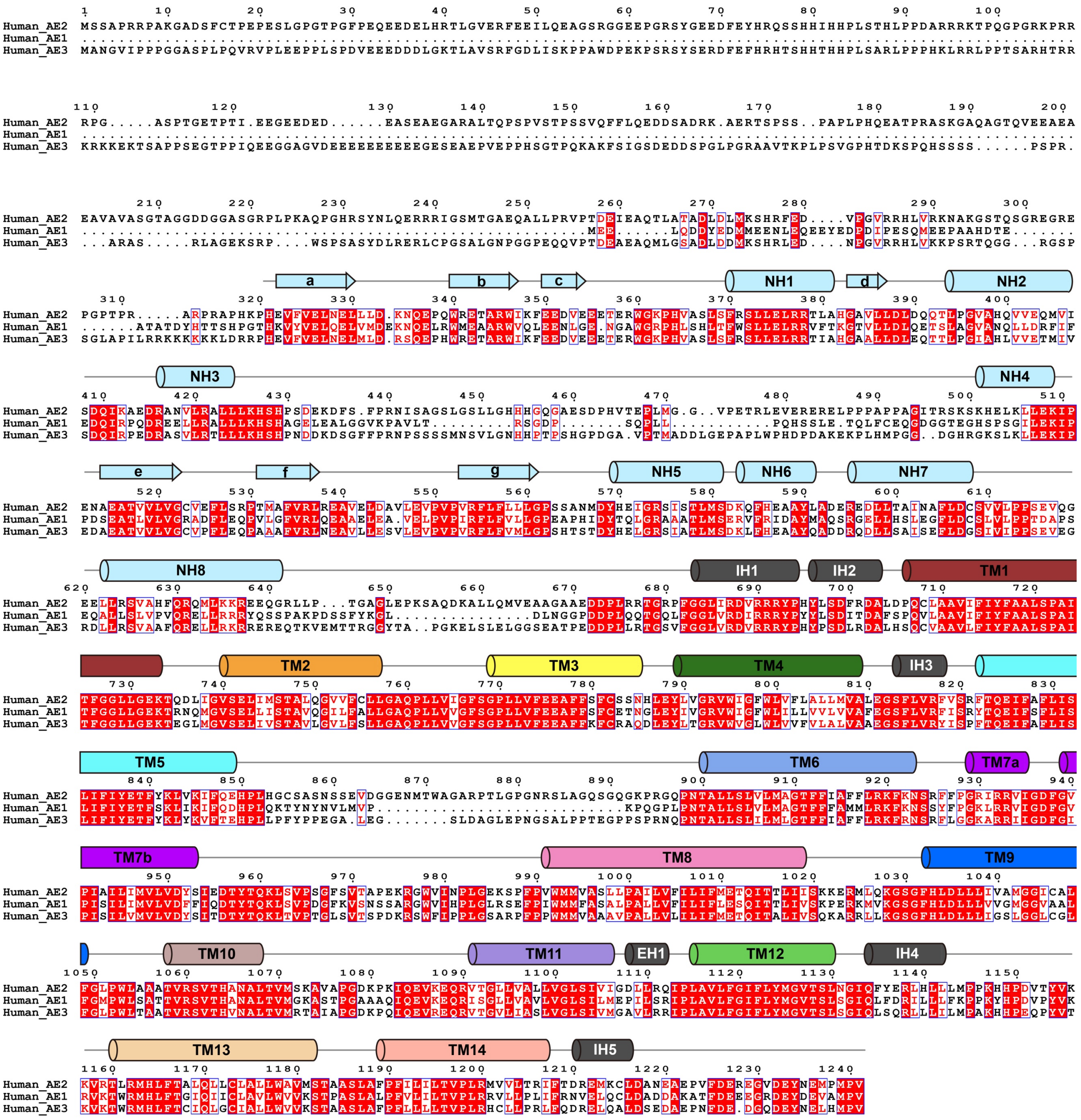

Supplementary Fig. 9. The amino acid sequence alignment among anion exchange protein AE1, AE2, and AE3 from *Homo sapiens*. The protein sequences of human AE2 (UniProt - P04920), human AE1 (UniProt - P02730), and human AE3 (UniProt - P48751) were aligned using the Clustal Omega server with the secondary structural elements of AE2 marked above the alignment. Residues are colored based on their conservation using the ESPript server, and the secondary structural elements of AE2 were colored the same as Fig 1d.

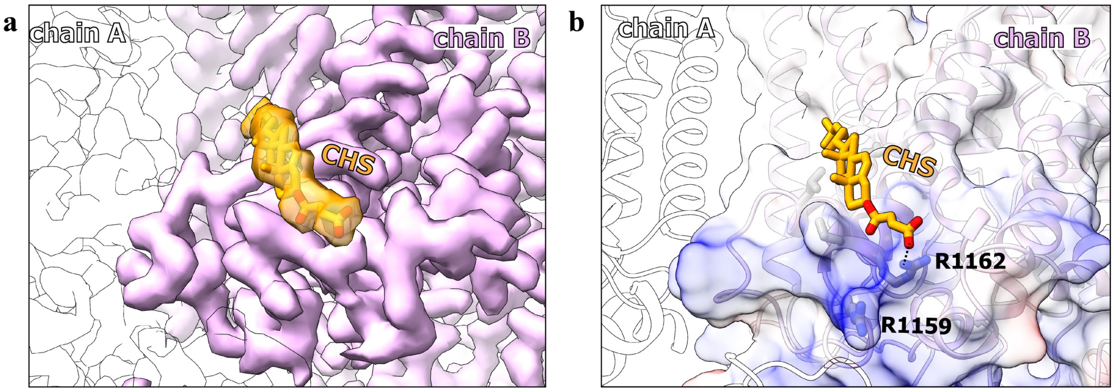

Supplementary Fig. 10. The cryo-EM density and the binding of CHS (cholesterol hemisuccinate). (a) The non-proteinous electron-density map (orange) in hAE2$^{acidic\text{-}KNO3}$ and the stick model of CHS modeled. **(b)** The cartoon model of the binding site of CHS. CHS was in orange and colored by elements.

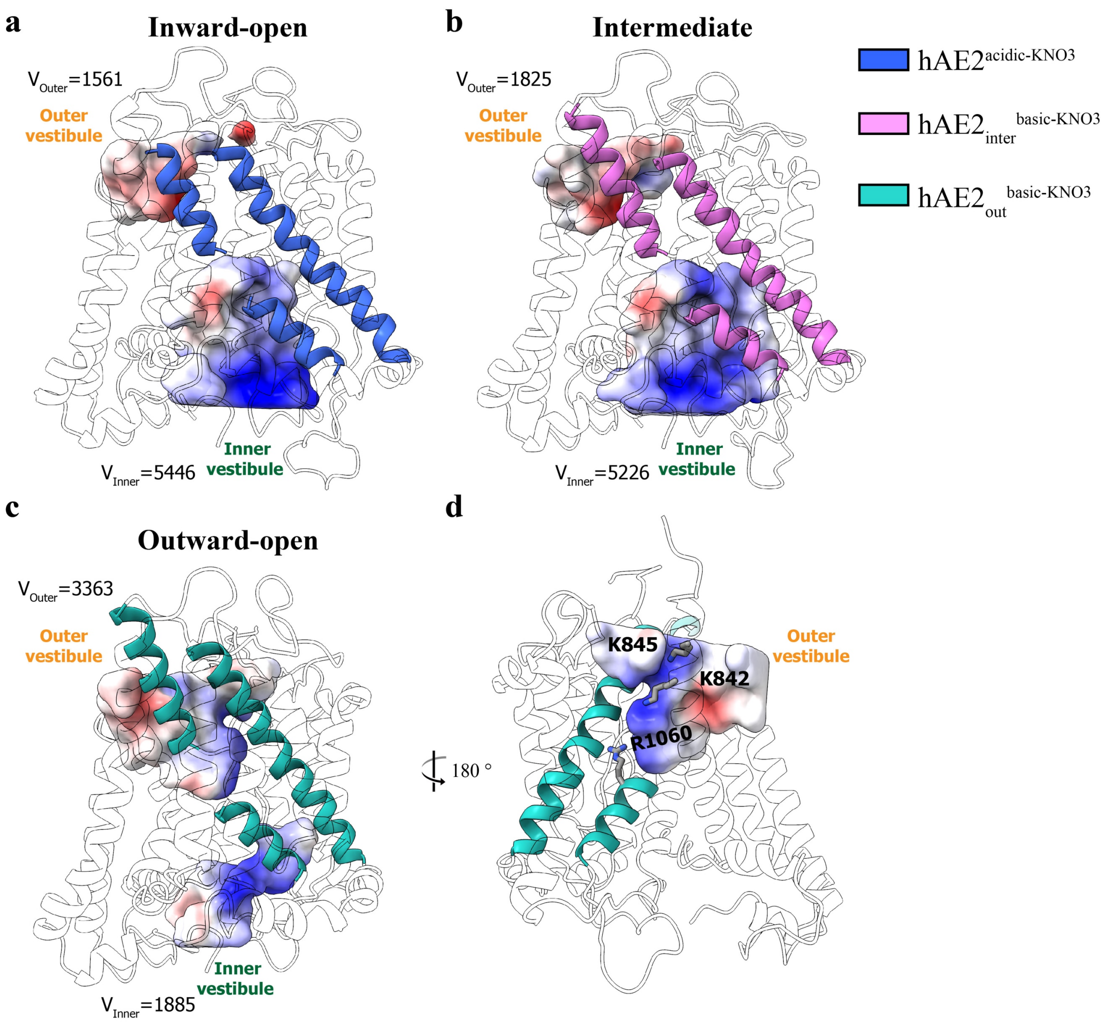

Supplementary Fig. 11. The inner vestibule and outer vestibule of hAE2 in the inward-open, intermediate, and outward-open conformations. (a) The large inner vestibule and the small outer vestibule exist in inward-open $hAE2^{acidic\text{-}KNO3}$. The volume of the inner vestibule is 5446 Å^{3,} and the volume of the outer vestibule is 1561 Å^3. **(b)** The large inner-vestibule and the small outer-vestibule exist in intermediate $hAE2_{inter}^{basic\text{-}KNO3}$. The volume of the inner vestibule is 5226 Å^{3,} and the volume of the outer vestibule is 1825 Å^3. **(c)** The small inner vestibule and the enlarged outer vestibule exist in intermediate $hAE2_{out}^{basic\text{-}KNO3}$. The volume of the inner vestibule is 1885 Å^{3,} and the volume of the outer vestibule is 3363 Å^3. **(d)** Proposed positively-charged entry/exiting routine for the anion substrates in the outer vestibule of outward-open $hAE2_{out}^{basic\text{-}KNO3}$.

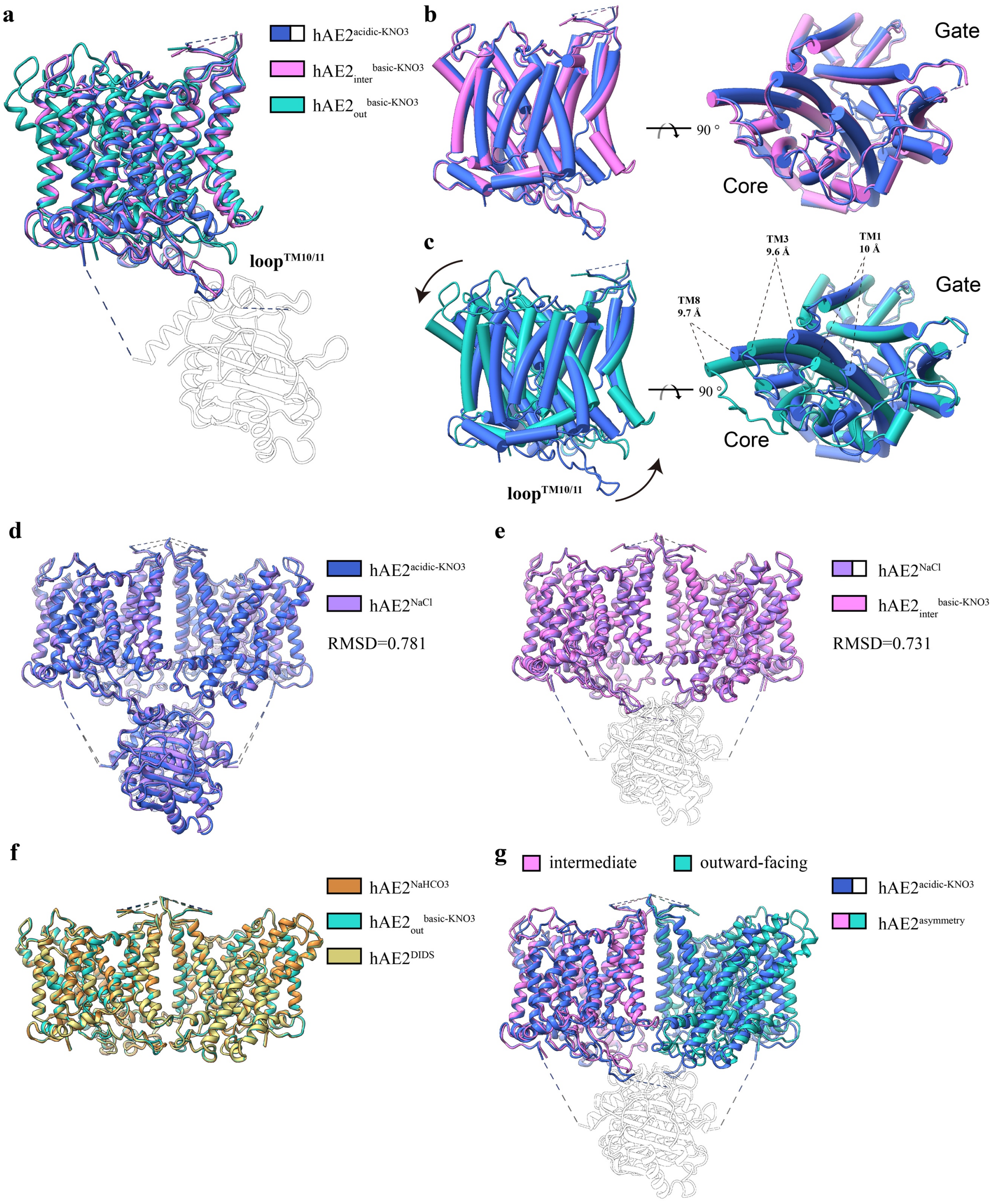

Supplementary Fig. 12. The structural superposition among the hAE2s in different states. (a) The overall structural superposition among the protomers of inward-facing conformation (blue), intermediate conformation (pink), and outward-facing conformation (green). **(b)** The structural superposition between $hAE2^{acidic\text{-}KNO3}$ and $hAE2_{inter}^{basic\text{-}KNO3}$ and the RMSD value is 1.245. The $loop^{CT}$ and $loop^{TM10/11}$ were excluded from the overall RMSD calculation. **(c)** The structural superposition between $hAE2^{acidic\text{-}KNO3}$ and $hAE2_{out}^{basic\text{-}KNO3}$ and the RMSD value is 5.002. The $loop^{CT}$ and $loop^{TM10/11}$ were excluded from the overall RMSD calculation. **(d)** The structural superposition between $hAE2^{acidic\text{-}KNO3}$ and $hAE2^{NaCl}$. The RMSD value is b 0.781 Å. **(e)** The structural superposition between $hAE2^{acidic\text{-}KNO3}$ and $hAE2_{inter}^{basic\text{-}KNO3}$. The overall RMSD is 0.731 Å. **(f)** The structural superposition among $hAE2^{NaHCO3}$

(orange), $hAE2_{out}^{basic\text{-}KNO3}$ (green), and $hAE2^{DIDS}$ (yellow). All are in an outward-facing conformation, and the RMSD between $hAE2_{out}^{basic\text{-}KNO3}$ and $hAE2^{NaHCO3}$ is 0.248, the RMSD between $hAE2_{out}^{basic\text{-}KNO3}$ and $hAE2^{DIDS}$ is 0.399 Å. **(g)** The structural comparison between $hAE2^{acidic\text{-}KNO3}$ and $hAE2^{asymmetry}$. The protomer in the intermediate conformation of $hAE2^{asymmetry}$ was colored in pink, and the promoter in the outward-facing conformation of $hAE2^{asymmetry}$ was colored in green.

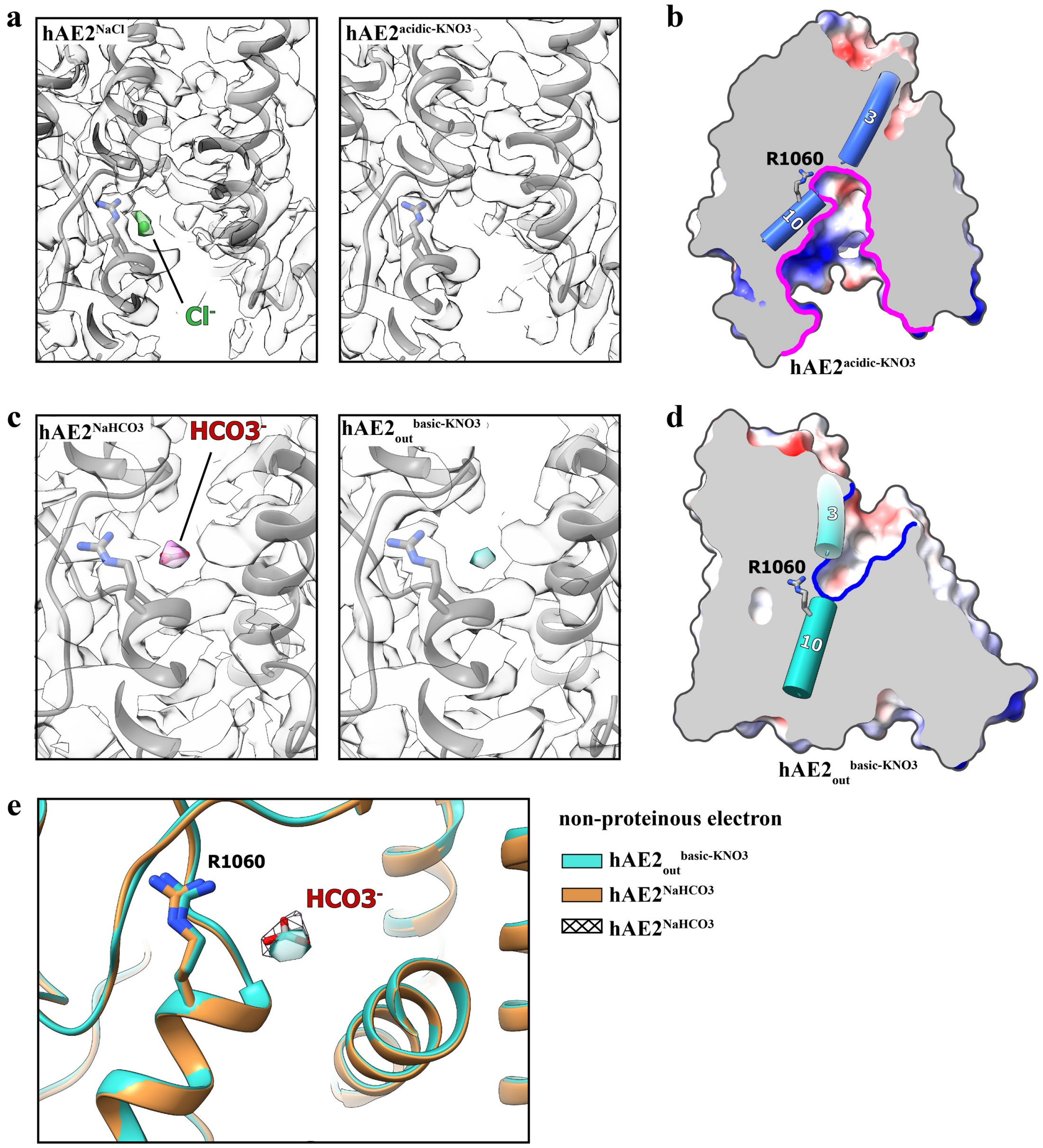

Supplementary Fig. 13. The anion binding pocket of hAE2$^{\text{acidic-KNO3}}$ and hAE2$_{\text{out}}^{\text{basic-KNO3}}$. (a) The cryo-EM density map of the anion binding pocket of hAE2^{NaCl} (left) and the surrounding region of hAE2$^{\text{acidic-KNO3}}$ (right). The electron density for Cl^- was shown as green surface. **(b)** The inner vestibule of hAE2$^{\text{acidic-KNO3}}$. The hAE2$^{\text{acidic-KNO3}}$ was shown as the solvent-accessible electrostatic surface potential maps and sliced to show the empty anion binding pocket, with the helices TM3 and 10 shown as blue cylinders and the inner vestibules delineated with pink lines. **(d)** The outer vestibule of hAE2$_{\text{out}}^{\text{basic-KNO3}}$. The hAE2$_{\text{out}}^{\text{basic-KNO3}}$ was shown as the solvent-accessible electrostatic surface potential maps and sliced to show the empty anion binding pocket, with the helices TM3 and 10 shown as cyan cylinders and the inner vestibules delineated with blue lines. **(c)** The cryo-EM density map of the anion binding pocket of hAE2$^{\text{NaHCO3}}$ (left) and the surrounding region of hAE2$_{\text{out}}^{\text{basic-KNO3}}$ (right). The electron density of HCO_3^- was shown as pink surface. (e) A comparison between the non-proteinous electron densities in hAE2$_{\text{out}}^{\text{basic-KNO3}}$ and hAE2$^{\text{NaHCO3}}$. The non-proteinous electron density of HCO_3^- was shown in mesh, while the non-

proteinous electron density in $hAE2_{out}^{basic-KNO3}$ was shown as green surface. Both electron density maps were contoured to the same level.

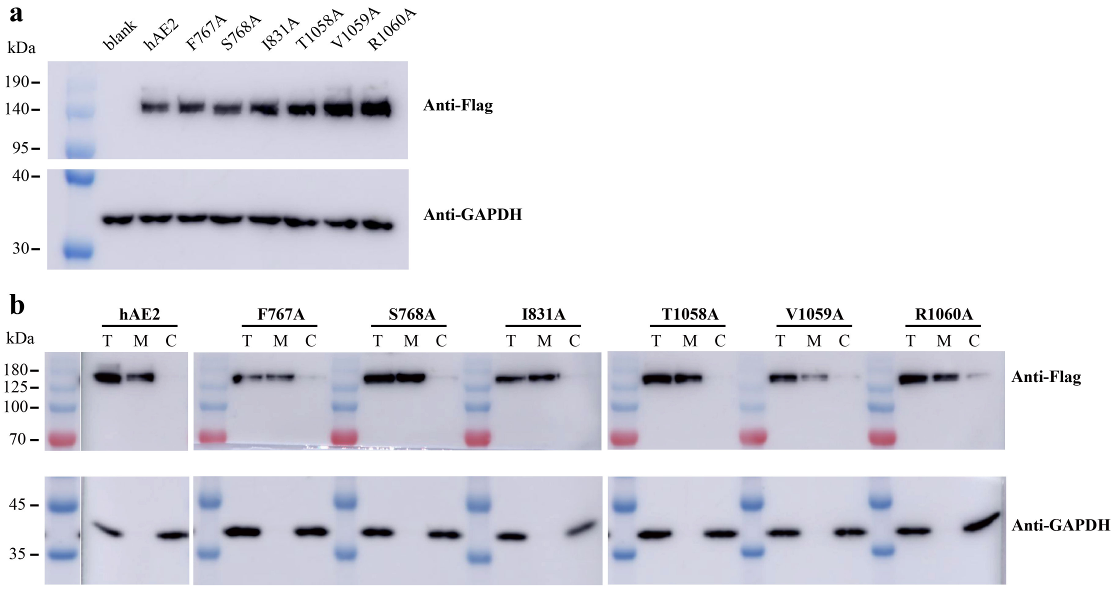

Supplementary Fig. 14. The analysis for the quantity of hAE2 and mutants using western blot. (a) The expression level of hAE2 and different mutants were detected using anti-Flag, and the cell quantity was normalized using anti-GAPDH. **(b)** The analysis for the membrane expression of hAE2 and mutants. The "T" above the picture represents the total protein, the "M" represents the membrane preparations, and the "C" represents the cytosol. The experiments were repeated three times with similar results.

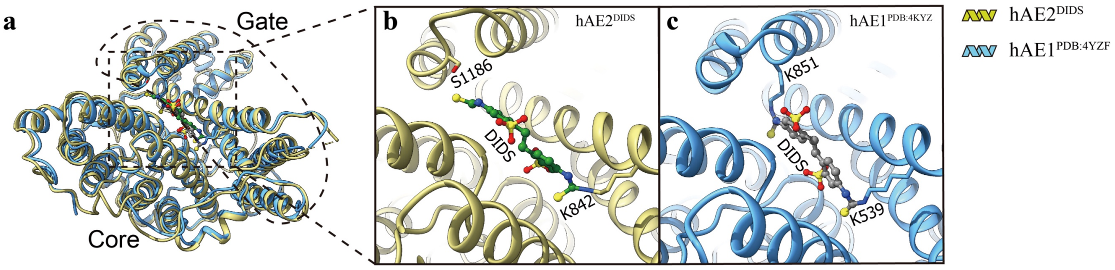

Supplementary Fig. 15. The structural comparison of DIDS bounded structures. (a) The model superposition of DIDS bounded hAE2 (hAE2^{DIDS}) and hAE1 (hAE1$^{PDB:4YZF}$). **(b) &(c)** Enlarged view of the DIDS-AE interactions.

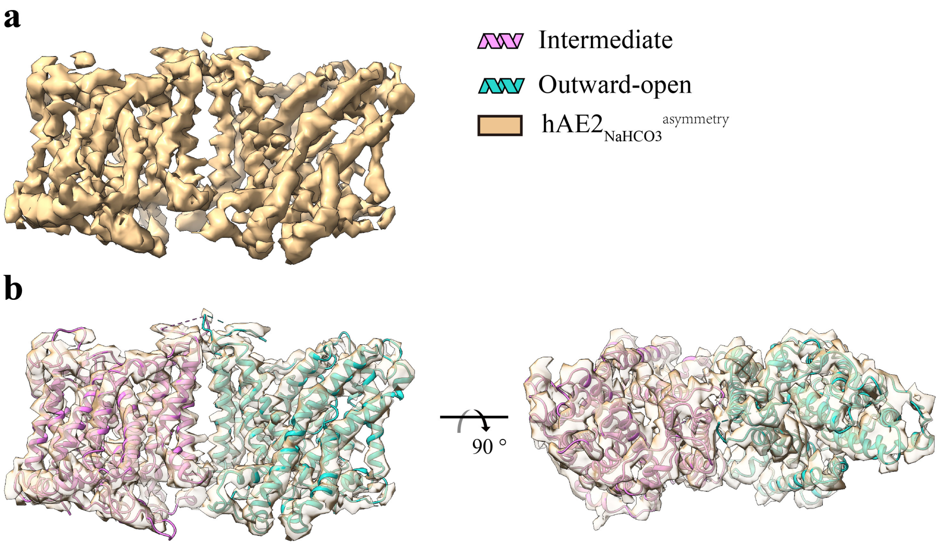

Supplementary Fig. 16. The asymmetric conformation of the hAE2 in HCO3--based buffer unsupplemented with DIDS (hAE2$_{NaHCO3}^{asymmetry}$). (a) The cryo-EM map of determined at an overall resolution of 4.6 Å. **(b)** The structural model of hAE2a^{symmetry} (supplemented with DIDS) fits well in the EM map of hAE2$_{NaHCO3}^{asymmetry}$.

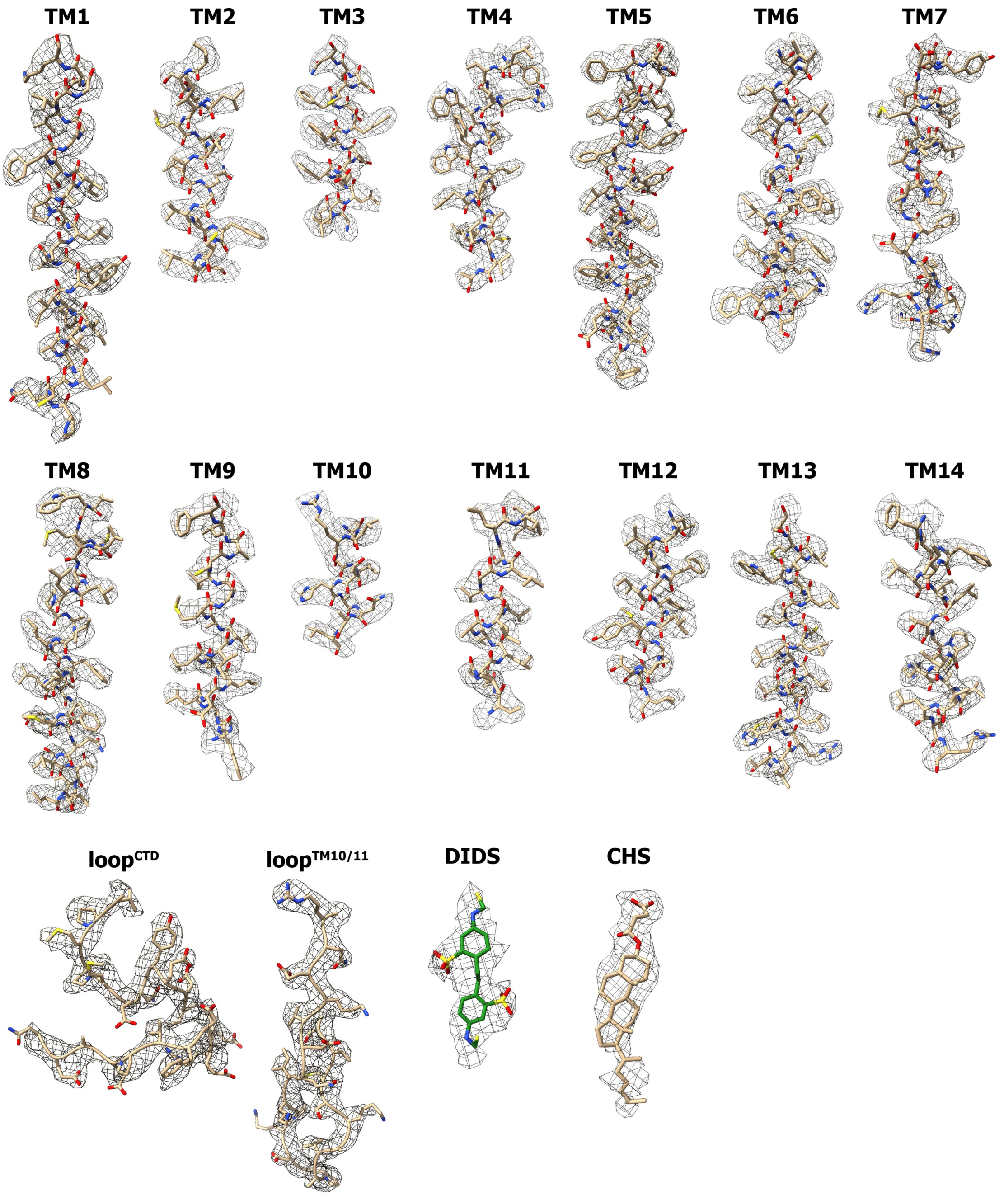

Supplementary Fig. 17. The cryo-EM density maps and the model-fitting of hAE2. The local density for the transmembrane helices, loop^{CT,} and loop$^{TM10/11}$ of hAE2$^{acidic\text{-}KNO3}$. The density maps for DIDS and CHS (cholesterol hemisuccinate) are shown at the same contour level as the protein structures.

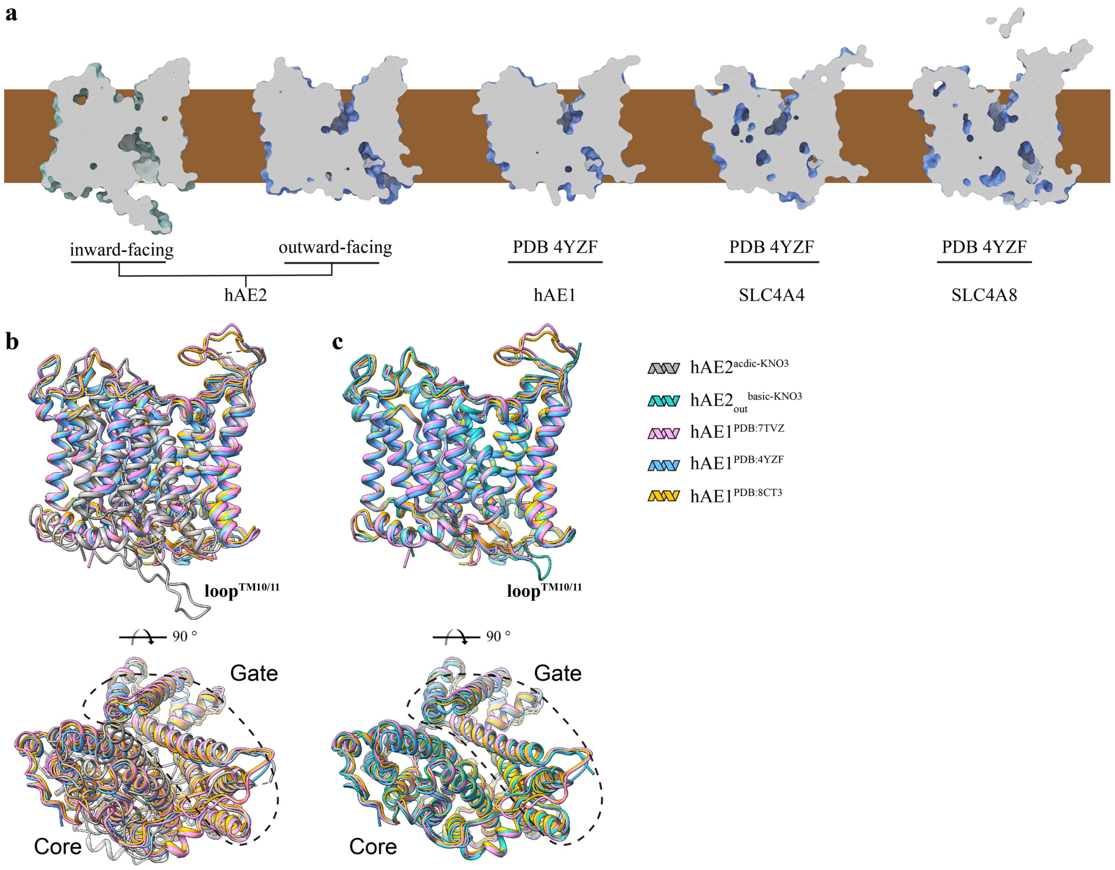

Supplementary Fig. 18. The model superposition of hAE2 with the reported structures of the SLC4A family. (a) A comparison among AE1, AE2, SLC4A4 and SLC4A8 structures. **(b)** The structural superposition of $hAE2^{acdic\text{-}KNO3}$ with the reported structures of hAE1 ($hAE1^{PDB:7TVZ}$, $hAE1^{PDB:4YZF}$, $hAE1^{PDB:8CT3}$). The RMSD value is 5.417 Å, 5.232 Å, and 5.513 Å, respectively. **(c)** The structural superposition of $hAE2_{out}^{basic\text{-}KNO3}$ with the reported structures of hAE1 (same with (b) and The RMSD value is 1.082 Å, 0.699 Å, 1.742 Å, respectively.

Supplementary Table 1. Cryo-EM data information

Data	Particles for final map reconstitution	Symmetry imposed	Resolution (Å)	monomer states	Presence of NTD	Presence of Loop^{CT}	pH
**AE2**$^{acidic\text{-}KNO3}$	230937	C2	3.32	Inward	yes	yes	7.25
**AE2**$^{basic\text{-}KNO3\text{-}Inter2}$	522410	C2	3.25	Intermediate	no	no	8.32
**AE2**$^{basic\text{-}KNO3\text{-}outopen}$	550035	C2	3.09	Outward	no	no	8.32
**AE2**^{NaCl}	678352	C2	3.06	Inward	yes	no	7.25
**AE2**^{NaHCO3}	652523	C2	2.89	Outward	no	no	7.25
**AE2**^{DIDS}	222365	C2	3.08	Outward	no	no	7.25
**AE2**^{asymmetry}	628875	C1	3.17	Intermediate /Outward	no	no	7.25

Supplementary Table 2-1.

Cryo-EM data collection, model refinement, and validation statistics.

	$AE2^{acidic\text{-}KNO3}$ (EMDB-34293) (PDB 8GVH)	$AE2_{out}^{basic\text{-}KNO3}$ (EMDB-34292) (PDB 8GVF)	$AE2_{inter}^{basic\text{-}KNO3}$ (EMDB-34289) (PDB 8GVA)
**Data collection and processing**			
Magnification	81000	81000	
Voltage (kV)	300	300	
Electron exposure ($e^-/Å^2$)	50	50	
Energy filter slit width	15 eV	15 eV	
Defocus range (μm)	1.0-2.6	1.3-2.7	
Pixel size (Å/pixel)	1.1	1.1	
Symmetry imposed	C2	C2	C2
Final particle images (no.)	3,057,925	3,107,433	3,107,433
Initial particle images (no.)	230,937	550,035	522,410
Map resolution (Å)	3.32	3.09	3.25
FSC threshold	0.143	0.143	0.143
Map resolution range (Å)	2.4-5.767	2.428-5.221	2.4-5.5
**Refinement**			
Initial model used (PDB code)	4YZF, 1HYN	8GVH	8GVH
Model resolution (Å)	3.3	3	3.2
FSC threshold	0.143	0.143	0.143
Map sharpening *B* factor ($Å^2$)	144.8	156.7	168.9
Model composition			
Non-hydrogen atoms	12602	8142	8142
Protein residues	1576	1022	1022
Ligands	Y01: 2	--	--
B factors ($Å^2$)			
Protein	99.45	40.43	67.11
Ligand	106.53	78.55	90.03
R.m.s. deviations			
Bond length (Å)	0.011	0.004	0.013
Bond angles (°)	0.77	0.544	0.861
Validation			
MolProbity score	1.69	1.52	1.62
Clashscore	6.17	4.6	5.08
Rotamer outliers (%)	0.07	0.34	0
Ramachandran plot			
Outlier (%)	0.13	0.3	0
Allowed (%)	5.58	3.85	5.03
Favored (%)	94.29	95.86	94.97

Supplementary Table 2-2.

Cryo-EM data collection, model refinement, and validation statistics.

	AE2^{NaCl} (EMDB-34288) (PDB 8GV9)	AE2^{NaHCO3} (EMDB-34290) (PDB 8GVC)	AE2^{DIDS} (EMDB-34287) (PDB 8GV8)	AE2^{asymmetry} (EMDB-34291) (PDB 8GVE)
**Data collection and processing**				
Magnification	81000	81000	81000	
Voltage (kV)	300	300	300	
Electron exposure (e$^-$/Å^2)	50	50	50	
Energy filter slit width	15 eV	15 eV	15 eV	
Defocus range (μm)	1.2-2.4	1.3-3.0	1.5-2.7	
Pixel size (Å/pixel)	1.1	1.1	1.1	
Symmetry imposed	C2	C2	C2	C1
Initial particle images (no.)	6,667,978	3,391,392	3,107,433	3,107,433
Final particle images (no.)	678,352	652,523	222,365	628,875
Map resolution (Å)	3.06	2.89	3.08	3.17
FSC threshold	0.143	0.143	0.143	0.143
Map resolution range (Å)	2.4-4.868	2.428-4.942	2.428-5.444	2.4-5.802
**Refinement**				
Initial model used (PDB code)	8GVH	8GVH	8GVH	8GVH
Model resolution (Å)	3	2.9	3	3.1
FSC threshold	0.143	0.143	0.143	0.143
Map sharpening *B* factor (Å^2)	149.9	149.3	140	153.8
Model composition				
Non-hydrogen atoms	12240	8150	8231	8124
Protein residues	1532	1022	1027	1020
Ligands	CL: 2	BCT: 2	4KU: 2	--
B factors (Å^2)				
Protein	54.84	47.15	43.89	47.94
Ligand	53.27	73.53	63.19	55.88
R.m.s. deviations				
Bond length (Å)	0.011	0.013	0.002	0.003
Bond angles (°)	0.818	0.748	0.611	0.655
Validation				
MolProbity score	1.6	1.78	1.29	1.73
Clashscore	4.85	3.77	3.73	6.17
Rotamer outliers (%)	0.45	0	0.23	0
Ramachandran plot				
Outlier (%)	0.2	0.2	0.1	0
Allowed (%)	4.75	3.16	2.55	5.73
Favored (%)	95.05	96.65	97.35	94.27

Uncropped figures

Supplementary Figure 1b

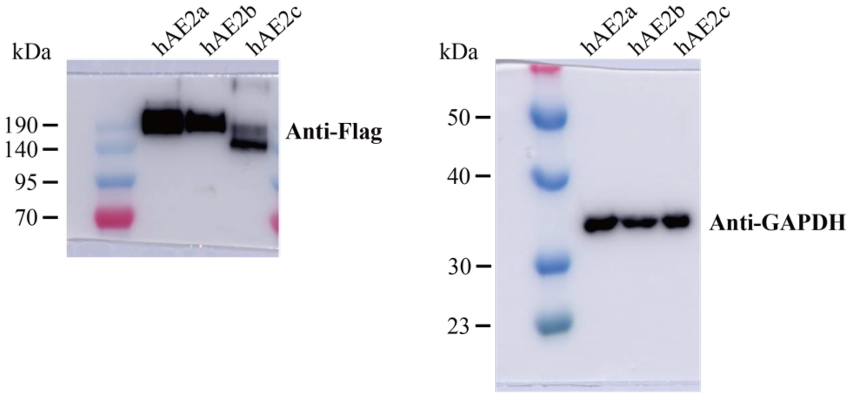

Supplementary Figure 1f

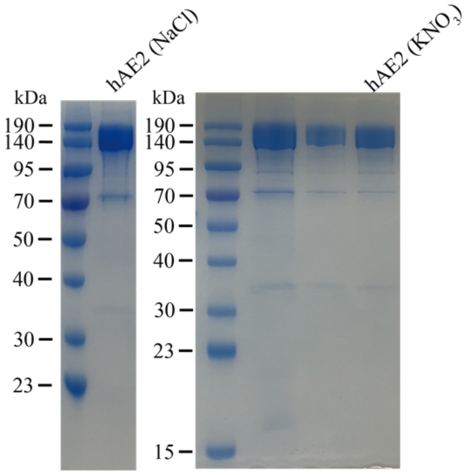

Supplementary Figure 14a

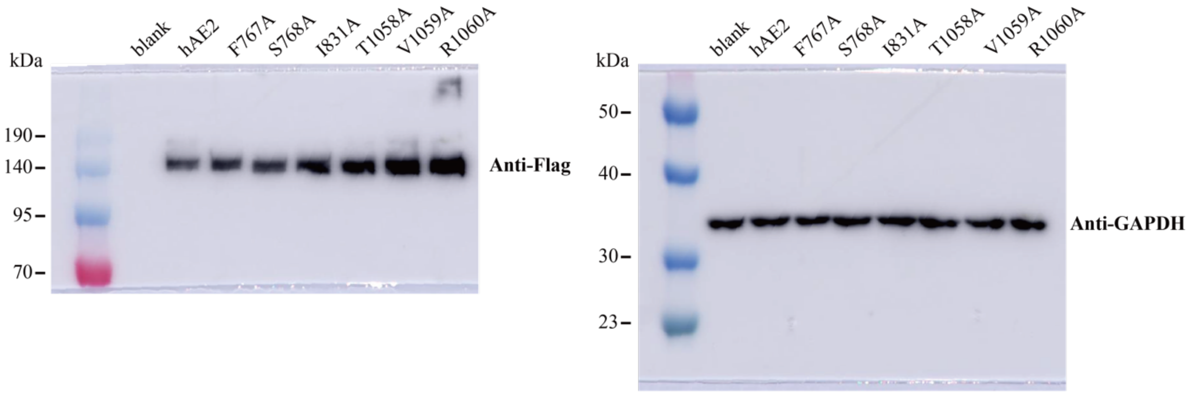

Supplementary Figure 14b

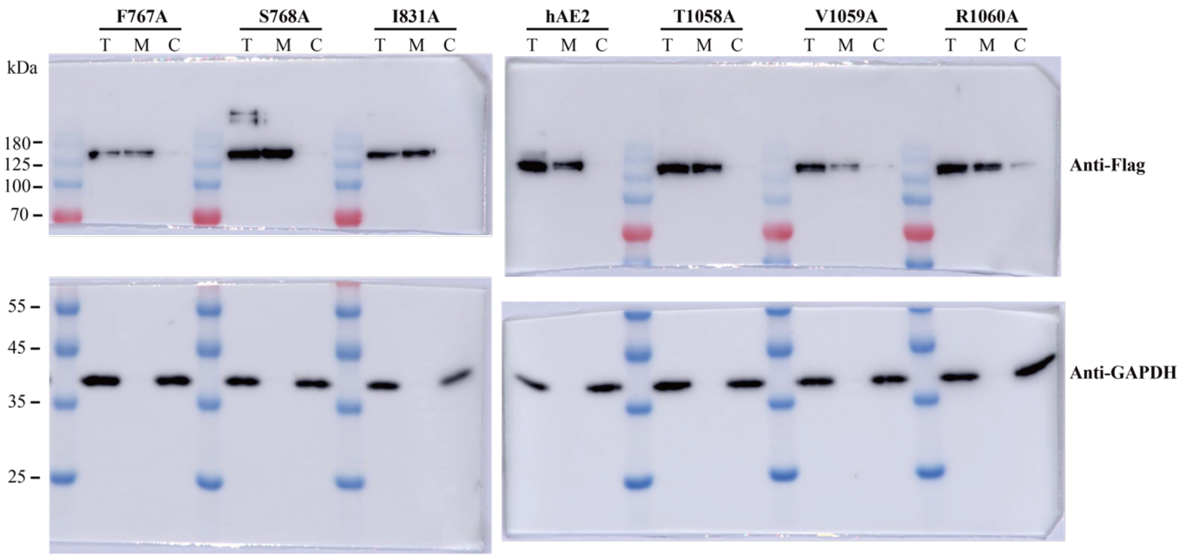
F767A
S768A
I831A
hAE2
T1058A
V1059A
R1060A
T M C
kDa
180
125
100
70
55
45
35
25
Anti-Flag
Anti-GAPDH

Primer List

hAE2a-F	CCGGCGCGCCATGAGCAGCGCCCCTCG
hAE2b-F	CCGGCGCGCCATGACTCAGCCAGAGCCAGAGAG
hAE2c-F	CCGGCGCGCCATGGGGAGGAAGACTTTGAGTACCACC
hAE2-R	CCGCGGCCGCCTACACAGGCATGGGCA
hAE2-F767A-F	GTGATCGGCGCCTCAGGGCCCCTGCTG
hAE2-F767A-R	GGGCCCTGAGGCGCCGATCACCAACAG
hAE2-S768A-F	GATCGGCTTCGCAGGGCCCCTGCTG
hAE2-S768A-R	GGGCCCTGCGAAGCCGATCACCAACAG
hAE2-I831A-F	GCCTTCTTGGCCTCACTCATCTTCATCTATGAGACC
hAE2-I831A-R	GATGAGTGAGGCCAAGAAGGCGAAGATCTCC
hAE2-T1058A-F	CTGCTGCCGCTGTCCGCTCTGTCACTC
hAE2-T1058A-R	AGCGGACAGCGGCAGCAGCCAACCAG
hAE2-V1059A-F	CTGCCACTGCCCGCTCTGTCACTCAC
hAE2-V1059A-R	CAGAGCGGGCAGTGGCAGCAGCC
hAE2-R1060A-F	CAGAGCGGGCAGTGGCAGCAGCC
hAE2-R1060A-R	GAGTGACAGAGGCGACAGTGGCAGCAGCC
