# Supplementary material for: The structural basis of the pH-homeostasis mediated by the Cl−/HCO3− exchanger, AE2
Source: Nat Commun. 2023 Mar 31;14:1812. doi: 10.1038/s41467-023-37557-y (PMC10066210; doi:10.1038/s41467-023-37557-y)
Supplement: Supplementary file 3 — Description of Additional Supplementary Files [file 41467_2023_37557_MOESM3_ESM.pdf]

Description of Additional Supplementary Files

File Name: Supplementary Movie 1
Description: The transporting process of HCO_3^-/Cl^- exchange by human AE2. The TMDs of dimeric hAE2 was shown as cartoon model and a dynamic process of the transportation was calculated based on the conformational changes among the cryo-EM structures determined. The gate domains were colored in gray, and the core domains were colored in blue.
