# Supplementary material for: The structural basis of the pH-homeostasis mediated by the Cl−/HCO3− exchanger, AE2
Source: Nat Commun. 2023 Mar 31;14:1812. doi: 10.1038/s41467-023-37557-y (PMC10066210; doi:10.1038/s41467-023-37557-y)
Supplement: Supplementary file 5 — Reporting Summary [file 41467_2023_37557_MOESM5_ESM.pdf]

nature portfolio

Corresponding author(s): Yu Cao

Last updated by author(s): Mar 15, 2023

Reporting Summary

Nature Portfolio wishes to improve the reproducibility of the work that we publish. This form provides structure for consistency and transparency in reporting. For further information on Nature Portfolio policies, see our Editorial Policies and the Editorial Policy Checklist.

Statistics

For all statistical analyses, confirm that the following items are present in the figure legend, table legend, main text, or Methods section.

n/a	Confirmed	
☐	☒	The exact sample size (*n*) for each experimental group/condition, given as a discrete number and unit of measurement
☒	☐	A statement on whether measurements were taken from distinct samples or whether the same sample was measured repeatedly
☒	☐	The statistical test(s) used AND whether they are one- or two-sided<br>*Only common tests should be described solely by name; describe more complex techniques in the Methods section.*
☒	☐	A description of all covariates tested
☒	☐	A description of any assumptions or corrections, such as tests of normality and adjustment for multiple comparisons
☐	☒	A full description of the statistical parameters including central tendency (e.g. means) or other basic estimates (e.g. regression coefficient) AND variation (e.g. standard deviation) or associated estimates of uncertainty (e.g. confidence intervals)
☒	☐	For null hypothesis testing, the test statistic (e.g. *F*, *t*, *r*) with confidence intervals, effect sizes, degrees of freedom and *P* value noted<br>*Give P values as exact values whenever suitable.*
☒	☐	For Bayesian analysis, information on the choice of priors and Markov chain Monte Carlo settings
☒	☐	For hierarchical and complex designs, identification of the appropriate level for tests and full reporting of outcomes
☒	☐	Estimates of effect sizes (e.g. Cohen's *d*, Pearson's *r*), indicating how they were calculated

Our web collection on statistics for biologists contains articles on many of the points above.

Software and code

Policy information about availability of computer code

Data collection	The cryo-EM data was collected by using EPU software (version 2.12.0.2771REL) on Titan Krios, equipment with K3 Summit direct electron detector (Gatan).
Data analysis	The EM data were processed using RELION-3..1.1 and cryoSPARC v3.2.2. The micrographs were Motion corrected using MotionCorr2_1.1.0. The structural model building and refinement were conducted using Coot version 0.9.5, BUSTER 2.10.4 and Phenix package 1.20.1_4487. For flow cytometry data process, we used FlowJo 10.0.7r2 and GraphPrism 9.0.0.

For manuscripts utilizing custom algorithms or software that are central to the research but not yet described in published literature, software must be made available to editors and reviewers. We strongly encourage code deposition in a community repository (e.g. GitHub). See the Nature Portfolio guidelines for submitting code & software for further information.

Data

Policy information about availability of data

All manuscripts must include a data availability statement. This statement should provide the following information, where applicable:

- Accession codes, unique identifiers, or web links for publicly available datasets
- A description of any restrictions on data availability
- For clinical datasets or third party data, please ensure that the statement adheres to our policy

The access number for human AE2 was UniProtKB – P04920 (https://www.uniprot.org/uniprotkb/P04920/entry). The coordinates data in this manuscript are

deposited at Protein Data Bank with accession codes: 8GVH, 8GVF, 8GVA, 8GV9, 8GVC, 8GV8, and 8GVE. The cryo-EM maps have been deposited in the Electron Microscopy Data Bank (EMDB) with accession codes EMD-34293, EMD-34292, EMD-34289 ,EMD-34288, EMD-34290, EMD-34287 and EMD-34291. We have already deposited the PDB and EMDB files to the database and the accession codes and accessible links were provided in the final manuscript.

Human research participants

Policy information about studies involving human research participants and Sex and Gender in Research.

Reporting on sex and gender	N/A
Population characteristics	N/A
Recruitment	N/A
Ethics oversight	N/A

Note that full information on the approval of the study protocol must also be provided in the manuscript.

Field-specific reporting

Please select the one below that is the best fit for your research. If you are not sure, read the appropriate sections before making your selection.

☒ Life sciences ☐ Behavioural & social sciences ☐ Ecological, evolutionary & environmental sciences

For a reference copy of the document with all sections, see nature.com/documents/nr-reporting-summary-flat.pdf

Life sciences study design

All studies must disclose on these points even when the disclosure is negative.

Sample size	The structural data for biological macromolecules are validated by physical and chemical laws, instead of statistics. For the enzymatic assay, three times repeat was applied to ensure the all the results all repeatable and reliable.
Data exclusions	For structural and functional experiments shown in the manuscript, all of the data were used.
Replication	For cryo-EM sample preparation, more than five times repeat was applied, we always can get valid data for analysis.
Randomization	For single particle analysis of EM, the particles were automatically picked. During 2D classification and 3D reconstruction, particles were allocated into experimental groups randomly. The resolution of the structures was calculated using gold-standard Fourier shell correlation.
Blinding	Blinding is not relevant to structural analysis because of the gold standard and related biochemical tests. All data collection and image processing procedures were automatically performed in unbiased manner which is generally used in the filed of cryo-EM.

Reporting for specific materials, systems and methods

We require information from authors about some types of materials, experimental systems and methods used in many studies. Here, indicate whether each material, system or method listed is relevant to your study. If you are not sure if a list item applies to your research, read the appropriate section before selecting a response.

Materials & experimental systems

n/a	Involved in the study	
☐	☒	Antibodies
☐	☒	Eukaryotic cell lines
☒	☐	Palaeontology and archaeology
☒	☐	Animals and other organisms
☒	☐	Clinical data
☒	☐	Dual use research of concern

Methods

n/a	Involved in the study	
☒	☐	ChIP-seq
☐	☒	Flow cytometry
☒	☐	MRI-based neuroimaging

Antibodies

Antibodies used	We used the commercial Mouse anti DDDDK-Tag mAb (ABclonal, AE005) to detect the expression of hAE2 and different mutants. The secondary antibody is Anti-mouse IgG, HRP-linked Antibody (Cell Signaling, #7076). We also use the GAPDH Rabbit mAb (Abclonal,

	Catalog number: A19056) to evaluate the cell quantity. The secondary antibody is Anti-rabbit IgG, HRP-linked Antibody (transgen, Catalog number: HS101-01). All the antibodies were diluted (1:10,000) by skim milk in TBST in prior to use.
Validation	validation statement available at the web page for each antibodies:<br>Mouse anti DDDDK-Tag mAb (ABclonal, AE005): https://abclonal.com/catalog-antibodies/MouseantiDDDDKTagmAb/AE005<br>Anti-mouse IgG, HRP-linked Antibody (Cell Signaling, #7076): https://www.cellsignal.cn/products/secondary-antibodies/anti-mouse-igg-hrp-linked-antibody/7076<br>GAPDH Rabbit mAb (Abclonal, Catalog number: A19056): https://abclonal.com/catalog-antibodies/GAPDHRabbitmAb/A19056<br>Anti-rabbit IgG, HRP-linked Antibody (transgen, Catalog number: HS101-01): https://www.transgen.com.cn/antibody_second/397.html

Eukaryotic cell lines

Policy information about cell lines and Sex and Gender in Research

Cell line source(s)	Expi293F cell line Thermofisher, A14528
Authentication	The cell lines are used to produce proteins for structural determination and analysis. It was purchased and the authentication is not conducted.
Mycoplasma contamination	No mycoplasma contamination was detected
Commonly misidentified lines<br>(See ICLAC register)	To our best knowledge, there is no commonly misidentified lines.

Flow Cytometry

Plots

Confirm that:

☐ The axis labels state the marker and fluorochrome used (e.g. CD4-FITC).

☐ The axis scales are clearly visible. Include numbers along axes only for bottom left plot of group (a 'group' is an analysis of identical markers).

☐ All plots are contour plots with outliers or pseudocolor plots.

☐ A numerical value for number of cells or percentage (with statistics) is provided.

Methodology

Sample preparation	Expi293 cells were incubated with fluorescent dye BCECF-AM and cells were subjected to the flow cytometry to measure the emission fluorescence intensities at 530 nm and 661 nm (BD LSRFortessa).
Instrument	BD LSRFortessa
Software	FlowJo
Cell population abundance	About 1 million per mL
Gating strategy	We detected the emission fluorescence intensities at 530 nm and 661 nm, then calculate the ration which can be used to calculate the intracellular pH.

☐ Tick this box to confirm that a figure exemplifying the gating strategy is provided in the Supplementary Information.
